# KLF12 transcriptionally regulates *PD‐L1* expression in non‐small cell lung cancer

**DOI:** 10.1002/1878-0261.13512

**Published:** 2023-09-02

**Authors:** Xiaohui Pan, Wenxin Zhang, Longsheng Wang, Hongjie Guo, Mingming Zheng, Honghai Wu, Qinjie Weng, Qiaojun He, Ling Ding, Bo Yang

**Affiliations:** ^1^ Zhejiang Province Key Laboratory of Anti‐Cancer Drug Research, Institute of Pharmacology and Toxicology, College of Pharmaceutical Sciences Zhejiang University Hangzhou China; ^2^ School of Pharmaceutical Science Wenzhou Medical University China; ^3^ The Innovation Institute for Artificial Intelligence in Medicine Zhejiang University Hangzhou China; ^4^ Cancer Center of Zhejiang University Hangzhou China

**Keywords:** histone acetylation, KLF12, NSCLC, PD‐L1, transcription

## Abstract

Recent studies have pointed to the role of Krüpple‐like factor 12 (KLF12) in cancer‐associated processes, including cancer proliferation, apoptosis, and metastasis. However, the role of KLF12 in tumor immunity remains obscure. Here, we found that KLF12 expression was significantly higher in non‐small cell lung cancer (NSCLC) cells with higher programmed death‐ligand 1 (*PD‐L1*) expression. Additionally, a positive correlation between KLF12 and PD‐L1 was observed in clinical patient tumor tissues. By chromatin immunoprecipitation (ChIP) analysis, KLF12 was identified to bind to the CACCC motif of the *PD‐L1* promoter. Overexpression of *KLF12* promoted *PD‐L1* transcription, whereas silencing of *KLF12* inhibited *PD‐L1* transcription. Furthermore, signal transducer and activator of transcription 1 (STAT1)‐ and STAT3‐triggered *PD‐L1* transcription was abolished in the absence of *KLF12*, and *KLF12* knockdown weakened the binding of STAT1 and STAT3 to the *PD‐L1* promoter. Mechanistically, KLF12 physically interacted with P300, a histone acetyltransferase. In addition, *KLF12* silencing reduced P300 binding to the *PD‐L1* promoter, which subsequently caused decreased acetylation of histone H3. *PD‐L1* transcription driven by *KLF12* overexpression was eliminated by *EP300* silencing. In immunocompetent mice, *KLF12* knockout inhibited tumor growth and promoted infiltration of CD8^+^ T cells. However, this phenomenon was not observed in immunodeficient mice. Overall, this study reveals KLF12‐mediated transcriptional regulation of *PD‐L1* in NSCLC; targeting KLF12 may be a potential therapeutic strategy for NSCLC.

## Graphical abstract

KLF12 binds to the *PD‐L1* promoter and promotes *PD‐L1* transcription. KLF12 recruits P300 to the *PD‐L1* promoter and maintains the acetylation of histone H3 (Ac‐H3). *KLF12* knockdown decreased Ac‐H3, which subsequently weakens the binding of STAT1 and STAT3 to the *PD‐L1* promoter. Inhibiting KLF12 may serve as a potential therapeutic strategy for NSCLC immunotherapy.

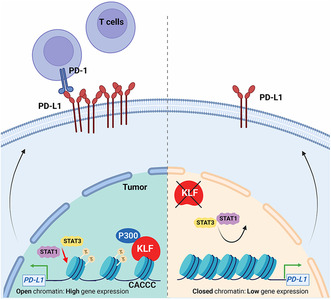

**Abbreviations**
Ac‐H3: acetylation of histone H3
ChIP: Chromatin immunoprecipitation
ICIs: immune checkpoint inhibitors
IHC: immunohistochemistry
KLF12: Krüpple‐like factor 12
KO: knock out
NSCLC: non‐small cell lung cancer
OS: overall survival
PD‐L1: programmed cell death ligand 1
PFS: progression‐free survival
q‐RT‐PCR: quantitative real‐time PCR
siRNA: short interfering RNA
STAT1: Signal transducer and activator of transcription 1
STAT3: Signal transducer and activator of transcription 3

## 1 Introduction

Krüpple‐like factor 12 (KLF12) belongs to the KLF family, a family of transcription factors with homologous DNA binding regions, which contain three highly conserved classical cysteine/histidine (Cys2/His2) zinc finger structures. This structure can bind to the “CACCC‐box” and “GC‐box” of DNA and exert transcriptional activation or repression [1, 2, 3]. KLF12 was originally discovered as a suppressor of the transcription factor AP‐2α, which is mainly expressed in tissues such as the brain, kidney, liver, and lung [2, 4] Recently, various studies have indicated that KLF12 is involved in tumor progression. KLF12 plays an important role in the malignant progression of poorly differentiated gastric cancer. Selective knockout of KLF12 can significantly induce growth retardation in the gastric cancer cell line HGC27 [5]. In colorectal cancer, KLF12 promotes tumor growth by directly activating early growth response protein (EGR1). The expression levels of KLF12 and EGR1 are associated with poor prognosis of the tumor and serve as potential diagnostic markers and therapeutic targets [6]. In addition, KLF12 has been shown to regulate the proliferation of natural killer cells in mice [7], which indicates that it may be involved in immunity. Nonetheless, the role of KLF12 in the regulation of antitumor immunity is still unknown.

Numerous studies have indicated that programmed cell death‐1 (PD‐1) and its ligand PD‐L1 play a key role in the immune escape of cancer cells by inactivating T‐cell function. It is generally accepted that PD‐L1 is highly expressed in tumors, and the binding of PD‐L1 to PD‐1 on T cells inhibits tumor immunity by suppressing the transcription of genes and cytokines required for T‐cell activation, enabling tumors to escape T‐cell surveillance. Based on this, blocking the PD‐1/PD‐L1 signaling pathway with immune checkpoint inhibitors (ICIs) restores immune surveillance and augments T‐cell‐mediated antitumor responses, which leads to the development of ICIs for tumor treatment. Compelling evidence in both preclinical and clinical studies holds great promise for ICIs in tumor treatment, and targeting PD‐L1 is related to a significant clinical response in a wide range of cancer patients. Additionally, small‐molecule inhibitors targeting PD‐L1 have shown strong antitumor activity in substantial preclinical studies. Thus, PD‐L1 has indeed proven to be an effective target in cancer therapy to augment antitumor immunity.

Multiple transcriptional regulatory mechanisms of PD‐L1 have been gradually unraveled [8]. *JAK1/2*, *KRAS*, and *EGFR* mutations have been shown to regulate the expression of PD‐L1 [9, 10, 11]. In addition, the expression of PD‐L1 is subject to epigenetic regulation, including histone acetylation and methylation [12]. In particular, bromodomain‐containing protein 4 (BRD4) and histone methyltransferase MLL1 are reported to transcriptionally enhance *PD‐L1* expression [13, 14]. However, histone deacetylase, enhancer of zeste homolog 2, and DNA methyltransferase 1 are involved in the decreased expression of PD‐L1 [15]. Many transcriptional regulators, including signal transducers and activators of transcription 1 (STAT1) and STAT3, Myc, IRF1, NF‐кB, hypoxia‐inducible factor‐1α, β‐catenin, and YAP1/TAZ, also contribute to promoting *PD‐L1* transcription in response to oncogenic signaling and other stimuli [8]. Nevertheless, the transcriptional regulation of *PD‐L1* varies under the currently known pathological conditions. For instance, MET expression/activation upregulates PD‐L1 expression in lung adenocarcinoma [16]. In addition, studies have also shown that MET mediates the decreased expression of PD‐L1 by activating GSK3β in liver cancer [17, 18]. Therefore, it is critical to further delineate the molecular mechanism of PD‐L1 regulation to develop effective strategies against cancer.

In this study, we found that KLF12 is significantly correlated with PD‐L1 expression in non‐small cell lung cancer (NSCLC) patient tumor tissues. Further analysis revealed that KLF12 binds to the *PD‐L1* promoter and promotes *PD‐L1* transcription. Furthermore, *KLF12* knockdown weakened STAT1‐ and STAT3‐triggered *PD‐L1* transcription. Mechanistically, *KLF12* knockdown reduces P300 recruitment to the *PD‐L1* promoter and further causes decreased acetylation of histone H3 (Ac‐H3). In immunocompetent mice, *KLF12* knockout increases the infiltration of CD8^+^ T cells and ultimately mediates tumor regression. Collectively, our research not only finds a novel transcription factor for PD‐L1 in NSCLC but also suggests that inhibition of KLF12 expression may be a potential therapeutic strategy for NSCLC.

## 2 Materials and methods

### 2.1 Antibodies and reagents

Anti‐KLF12 (#ab129459), Anti‐Histone H3 (acetyl K27) (#ab4729), Anti‐Histone H3 (acetyl K18) (#ab1191), Anti‐Histone H3 (acetyl K14) (#ab52946) was purchased from Abcam (Cambridge, UK). Anti‐PD‐L1 (#13684S), Anti‐Phospho‐STAT1 (#7649), Anti‐Phospho‐STAT3 (#7649), Anti‐P300 (#54062), and Anti‐CBP (#7389) were purchased from Cell Signaling Technology (Danvers, MA, USA). Anti‐β‐actin (#sc‐1615) and Anti‐PCAF (#sc‐13 124) were purchased from Santa Cruz Biotechnology (Dallas, TX, USA). Anti‐STAT1 (#10144‐2‐AP) and Anti‐STAT3 (#10253‐2‐AP), anti‐KLF12 (#13156‐1‐AP) were purchased from Proteintech (Chicago, IL, USA). Anti‐PD‐L1 (#UM800121) was from Origene (Rockville, MD, USA). PE‐conjugated anti‐human CD274 antibody (#557924), FITC‐conjugated CD274 antibody (#558065), FITC‐conjugated CD47 (#556045), PE‐conjugated anti‐human CD273 antibody (#558066), and PE‐conjugated mouse normal IgG (#555749) were purchased from BD Biosciences (San Jose, CA, USA). FITC Rat IgG2b κ isotype control (#400605), FITC Rat IgG2a κ isotype control (#400506), PE Rat IgG2a λ isotype control (#400635), FITC‐conjugated anti‐mouse CD3 (#100204), FITC‐conjugated anti‐mouse CD8a (#100705), PE‐conjugated anti‐mouse CD45 (#103106), and FITC anti‐human MHC I (#343303) were purchased from Biolegend (Chicago, IL, USA). The siRNA‐negative control and sequence‐specific short RNA duplex pools for interference were purchased from GenePharma (Suzhou, China). Recombinant Human IFNγ was obtained from SinoBiological (11725‐HNAS, Beijing, China). Protein synthesis inhibitor cycloheximide (CHX, #HY‐12320) was purchased from Med Chem Express (Monmouth Junction, NJ, USA). Olaparib (T3015) and SGC‐CBP30 (T6668), C646 (T2452) were purchased from TargetMol (Shanghai, China).

### 2.2 Cell culture

All the cell lines were obtained from Cell Bank of Shanghai Institutes for Biological Sciences, Chinese Academy of Sciences (Shanghai, China) and were authenticated via short tandem repeat (STR) profiling, most recent authentication on September 15, 2020. NCI‐H460 (RRID: CVCL_0459), NCI‐H1299 (RRID: CVCL_0060), NCI‐H1975 (RRID: CVCL_1511), and CT26 (RRID: CVCL_7254) was maintained in RPMI‐1640 (#31800; Gibco, Grand Island, NY, USA) with 10% fetal bovine serum (FBS) (SV30160.03; Hyclone, Logan, UT, USA). A549 (RRID: CVCL_0023) was maintained in F12 (#N670; Sigma, St. Louis, MO, USA) and 10% FBS. HEK293T (RRID: CVCL_0063) was cultured in DMEM (Gibco, #12800) with 10% FBS. All the media contained 1% penicillin/streptomycin (Gibco, #15140122), and all cells were maintained at 37 °C in a 5% CO_2_ incubator. All cell lines were performed and verified to be free of mycoplasma.

### 2.3 Cell sorting

After incubation for 12 h, NCI‐H460 and NCI‐H1299 cells were digested by trypsin (Keyi, China) and collected, washed twice with phosphate‐buffered saline (PBS), and blocked by 3% bovine serum albumin (BSA, diluted by PBS) at room temperature for 45 min. After washing with PBS, the cells were then stained with PE‐conjugated CD274 or PE‐conjugated mouse normal IgG and incubated at room temperature for 2 h (5 μL/2 × 10^5^ cells in 100 μL 0.2% BSA). After washing with PBS, cells were resuspended in 0.5 mL of PBS and sorted using BD FACS AriaII (BD Biosciences, San Jose, CA, USA) flow cytometer. The PD‐L1 expression was determined by relative fluorescence intensity (RFI) against PE‐conjugated mouse normal IgG, the cells with RFI of 10^2^ were regarded as PD‐L1 nonexpression cells, and the cells with RFI of 10^4^ were regarded as PD‐L1 high expression cells. The above experiments were performed under aseptic conditions.

### 2.4 Flow cytometry

Cells were washed twice with PBS, and blocked with 3% BSA at room temperature for 45 min. After washing with PBS, the cells were then stained with PE‐conjugated CD274 or PE‐conjugated mouse normal IgG and incubated at room temperature for 2 h. After washing with PBS, samples were measured by BD FACSuite™ flow cytometry (BD Bioscience). For tumor tissues, tissues were separated into single cells with tissue dissociator (Miltenyi Biotec, San Jose, CA, USA). Then, the cells were stained with anti‐CD3, anti‐CD8a, and anti‐CD45 at room temperature for 30 min, and with anti‐PD‐L1 for 2 h (1 μL/2 × 10^5^ cells). After washing with PBS, samples were measured by BD FACSuiteTM flow cytometry.

### 2.5 Gene‐Chip analysis

After verification of the expression of PD‐L1, PD‐L1 expression, and nonexpression cells were sent for whole‐genome gene chip analysis to screen differential gene expression. The gene chip analysis was performed by SBC Biomedlab (Shanghai, China). And the differential genes were further investigated by Genecards (Table S1).

### 2.6 Plasmid construction

The 2 kb *PD‐L1* promoter (#P7914) luciferase plasmid was purchased from MiaoLingPlasmid (Wuhan, China). The vector pcDNA3.0 was purchased from Invitrogen of Thermofisher (Waltham, MA, USA). The KLF12 was obtained from polymerase chain reaction (PCR) amplification with KOD‐Plus‐Neo kit (Toyobo, #KOD‐401, Japan) according to the manufacturer's instructions. The specific primers are as follows: KLF12: forward, 5′‐AGTCAGGGATCCATGAATATCCATATGAAGAGGAAAAC‐3′, reverse, 5′‐AGTCAGGAATTCTCACACCAACATATGCCTCCGGC‐3′.

### 2.7 Short interfering RNA (siRNA)‐mediated silencing

Cells were seeded in six‐well plates for each well and allowed to grow for 12 h. Then, cells were transfected with transfection reagent JetPRIME (#114‐15; Polyplus, France), Jet PRIME Buffer (Polyplus, #712‐60, Illkirch, France) and *KLF12* (*CD274*, *EP300*, *CREBBP*, and *KAT2B*) siRNA or scrambled siRNA (siRNA‐negative control, NC) for 24 h or 48 h. The transfection system was as follows: 200 μL JetPRIME Buffer (Polyplus, #712‐60), 2 μL JetPRIME and 2.5 μL siRNA (20 μm). The siRNA sequences are shown in Table S2.

### 2.8 RNA isolation and quantitative real‐time PCR (q‐RT‐PCR)

Total mRNA was collected and extracted with TRIzol reagent (Takara, #9109, Tokyo, Japan), and RNA was quantified using the NanoDrop Spectrophotometer ND‐1000 (Thermo Fisher Scientific, Waltham, MA, USA). cDNA was synthesized using TransScript One‐Step gDNA Removal and cDNA Synthesis SuperMix (#AT311‐03; TransGen Biotech, Beijing, China). Quantitative‐real time‐PCR was performed on 7500 Fast Dx Real‐Time PCR Instrument (Applied Biosystems, Singapore City, Singapore). Fold changes in the expression of each gene were calculated by a comparative threshold cycle (Ct) method. All reactions were performed in duplicate and beta‐actin was used as the normalizing gene. The primers used are listed in Table S3.

### 2.9 Western blot

Cells were lysed in 1% NP‐40 lysis buffer (25 mmol·L^−1^ Tris‐base, pH 7.4, 150 mmol·L^−1^ NaCl, 10% glycerol). Then cells were separated by SDS/PAGE, transferred to PVDF membranes (Immobilon, #IPVH00010), blocked in TBST (150 mm NaCl, 10 mm Tris–HCl, pH 7.5, and 0.1% Tween 20) containing 5% non‐fat dry milk for 1 h, and incubated with the indicated antibodies overnight. Next, cells were treated with HRP‐conjugated secondary antibodies at a dilution of 1 : 5000. The specific bands were analyzed by chemiluminescence imaging system (Amersham Imager 600, #66210336; GE Healthcare, Little Chalfont, Buckinghamshire, UK).

### 2.10 Luciferase reporter assays

The cells were transfected with pGL4.14‐PD‐L1, Renilla, and *KLF12*‐HA or *KLF12* siRNA by using transfection reagent. The Renilla‐luciferase‐expressing plasmid (#E2231; Promega, Madison, WI, USA) was used as the internal control to normalize the transfection efficiency. The luciferase activity was detected using the Dual‐Luciferase Reporter Assay System (DL101‐01; Vazyme, Nanjing, China) following the manufacturer's protocol. The results are determined with relative luciferase activity (firefly luciferase/renilla luciferase).

### 2.11 Chromatin immunoprecipitation (ChIP) assay

Chromatin immunoprecipitation assays were carried out using the EZ ChIP Kit (Millipore, Billerica, MA, USA) according to the manufacturer's instructions. In brief, cells were cross‐linked with 37% formaldehyde and quenched with Glycine. After washing twice with cold PBS, cells were scraped and resuspended in lysis buffer. The DNA was sheared to 200–1000 base pairs in length by sonication. The supernatants were incubated overnight with indicated antibodies (KLF12, STAT1, STAT3, P300, H3K27, H3K18, and H3K14), negative control IgG, and positive control RNA polymerase II (Pol II). Next, Protein G Agarose was added to each IP and incubated for 2 h at 4 °C with rotation. After washing with wash buffer, the agarose was collected and reversed cross‐links to free DNA with NaCl at 65 °C overnight. The DNA was purified using spin columns and quantified by qPCR and q‐RT‐PCR. The primers are listed in Table S3.

### 2.12 CRISPR/Cas9‐mediated *Klf12* and *Klf8* knockout (KO)

The guide RNA sequences GCACTATTGTTGTACCGCTCC or TATCACTTGGAAACTCCAGT targeting murine *Klf12* or *Klf8*, respectively, were designed with CRISPR/Cas9 system (http://crispor.tefor.net/) and inserted into PX458 plasmid (#48138, Addgene, Watertown, MA, USA). CT26 cells were planted in 5‐cm dishes and transfected with PX458 and *Klf12*, or *Klf8* KO plasmid by using transfection reagent. Single cells were sorted into each well of 96‐well plates by fluorescence‐activated cell sorting (FACSAria II, BD). After 2 weeks of culture, cells were collected and KO efficiency was determined by immunoblot analysis.

### 2.13 Database analysis

The online database Gene Expression Profiling Interactive Analysis (GEPIA 2) was used to analyze the correlation of KLF family proteins and *PD‐L1* (gene name of PD‐L1) mRNA in NSCLC. The correlation between KLF12 expression and survival in NSCLC was analyzed by the PrognoScan database.

### 2.14 Immunohistochemistry (IHC)

All tissue samples were collected in compliance with the informed consent policy, and patients were enrolled at the Institutional Review Board at Zhejiang Cancer Hospital Committee in Hangzhou (China) from January 2019 to October 2022. The study was conducted in accordance with the Declaration of Helsinki and was approved by the Institutional Review Board at Zhejiang Cancer Hospital Committee in Hangzhou (China), and all patients provided written informed consent (IRB‐2019‐175 and IRB‐2022‐540).

Immunohistochemistry assay performed to detect PD‐L1 expression was mainly assessed on tumor cells, as we selected tumor cell subsets for evaluation. For immunohistochemical staining, all tissue specimens were incubated with 3% hydrogen peroxide (PV‐6001, ZSGB‐BIO, Beijing, China) after deparaffinization and then were blocked by incubating in blocking buffer (5% goat serum in PBS) for 30 min. Tissue specimens were incubated overnight with the anti‐KLF12 (Proteintech, #13156‐1‐AP) and anti‐PD‐L1 (Origene, #UM800121), followed by treatment with HRP‐conjugated secondary antibodies (PV‐6001/2, ZSGB‐BIO) and horseradish peroxidase‐conjugated avidin and were visualized with 3,3‐diaminobenzidine (DAB) (ZSBG Bio, #ZLI‐9018) following the manufacturer's protocol. The intensity and percentage of protein expression were analyzed by scoring on scale of “–” to “+++” with the image‐pro plus 6.0 software (Media Cybernetics, Inc., Rockville, MD, USA). (−: no staining, +: weak staining, ++: moderate staining, +++: high staining) and proportion (0–1%, 1%–24%, 25–50%, > 50%). The HistoScore more than 50% was considered high expression and less than 50% was considered low expression [19].

### 2.15 Animal experiment

Animal experiments were carried out under the guidelines approved by the Institutional Animal Care and Use Committee of Zhejiang University in Hangzhou (IACUC no.19‐136, 19‐262). Male 5‐week‐old BALB/c and BALB/c nude mice were purchased from Beijing Vital River Laboratory Animal Technology Co., Ltd. Mice were bred and maintained under specific pathogen‐free conditions and were provided sterilized food and water *ad libitum* at the Center for Drug Safety Evaluation and Research of Zhejiang University. For the immunocompetent mouse model, *Klf12*‐KO CT26 cells (5 × 10^5^), *Klf8*‐KO CT26 cells (5 × 10^5^), and their CTRL group were injected subcutaneously into 5‐week‐old BALB/c male mice. Tumor volumes were measured and recorded daily until the tumor reached a volume of 2000 mm^3^. After experiment, tumor tissues were sheared to single cells and incubated with antibodies for FACS analysis. Tumor tissues were homogenized, and the KLF12, KLF8, and PD‐L1 expression was analyzed by immunoblot analysis or q‐RT‐PCR. For the immunodeficient mouse model, *Klf12*‐KO CT26 cells (5 × 10^5^) and their CTRL group were injected subcutaneously into 5‐week‐old BALB/c nude mice. Tumor volumes were measured and recorded daily. After experiment, tumor tissues were homogenized, and the KLF12 and PD‐L1 expression was analyzed by immunoblot analysis.

### 2.16 Lentiviral production and transduction

The lentiviral expression of shRNA: shKLF12 or nontargeting control shRNA was cloned into the vector pLKO.1 and was constructed using the primers listed below. Lentiviral particles were produced by transfecting HEK293FT cells with pCMV‐R8.91 (packaging vector), pMD2‐VSVG (envelope vector), and shRNA plasmids by Lipofectamine 2000 (#11668019, Invitrogen) as previously described [19] [Canagliflozin primes antitumor immunity by triggering PD‐L1 degradation in endocytic recycling]. For infection, NCI‐H460 cells with 30–40% confluency in six‐well plates were supplemented with 1 mL lentiviral particles and 1 μL polybrene (6 mg·mL^−1^) for 16 h, and then incubated for 72 h after changing the medium.

shRNA targeted sequences are listed here: human *KLF12* forward: CCGGGTAGATCACTTCCAAACACTCGAGTGTTTGGAAGTGATCTACTTTTTG; human *KLF12* reverse: AATTCAAAAAGTAGATCACTTCCAAACACTCGAGTGTTTGGAAGTGATCTAC.

### 2.17 Statistical analysis

All of the statistical data are presented as the mean ± SD. Student's *t*‐test (two groups) and one‐way ANOVA with Dunnett's *post hoc* test (more than two groups) were used to determine statistical differences. ***, *P* < 0.001; **, *P* < 0.01; *, *P* < 0.05; n.s.: not significant. The statistical analysis was analyzed by the Prism 7 software (GraphPad Software Inc., La Jolla, CA, USA). For western blot, the densitometric analysis was performed by Quantity One 4.6 (Bio‐Rad, Hercules, CA, USA). For IHC analysis, the *P*‐value was calculated using Pearson's correlation test, in which *P* < 0.05 shows statistical significance.

## 3 Results

### 3.1 KLF12 is positively correlated with PD‐L1 in NSCLC

**Fig. 1:**
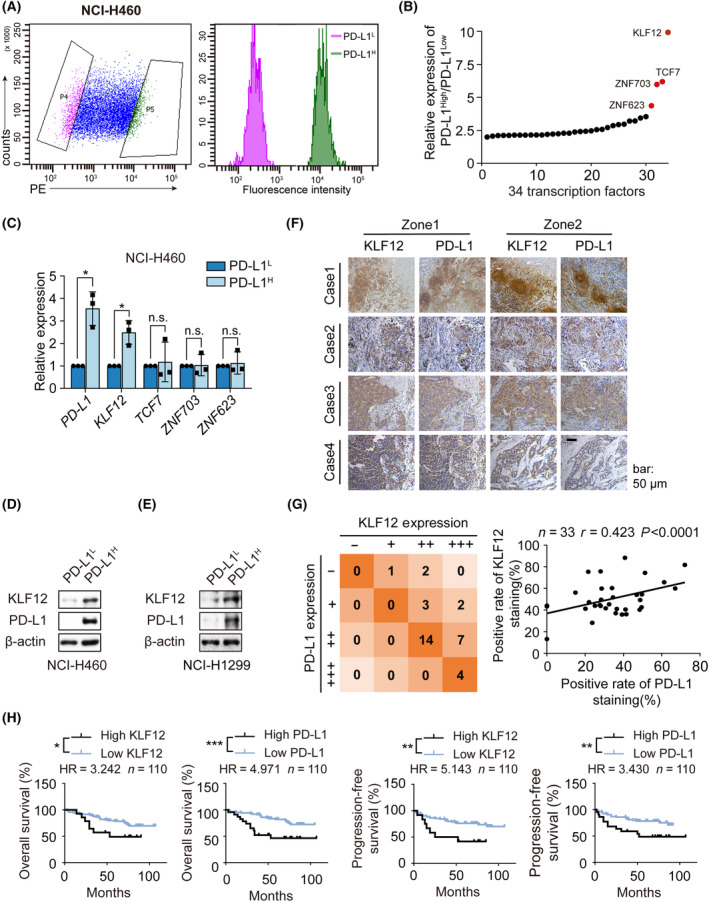
KLF12 correlated with PD‐L1 upregulation in NSCLC. (A) NCI‐H460 were gathered and labeled with PE anti‐human PD‐L1antibody, cells with low PD‐L1 membrane surface expression (relative fluorescence intensity was about 10^2^) were gated by gate P4 (violet), and cells with high PD‐L1 membrane surface (relative fluorescence intensity was about 10^4^) expression were gated by gate P5 (green). The two groups of cells were sorted and gathered by flow cytometry under aseptic conditions, then cells were sent for gene chip analysis (*n* = 2). (B) The differential expression of transcription regulation protein‐coding genes in PD‐L1^high^ subpopulation relative to PD‐L1^low^ subpopulation (*n* = 34). (C) The mRNA of the sorted NCI‐H460 cells with high or low PD‐L1 membrane expression was extracted and the transcription levels of *PD‐L1*, Krüpple‐like factor 12 (*KLF12*), *TCF7*, *ZNF703*, and *ZNF623* were detected by q‐RT‐PCR. The experiments were performed in triplicate. (D, E) The protein expression of PD‐L1 and KLF12 of the sorted NCI‐H460 cells (D) and NCI‐H1299 cells (E) with high or low PD‐L1 membrane expression was detected by western blot (*n* = 2). (F) The representative images of IHC staining of PD‐L1 or KLF12 expression in 33 NSCLC patients' tissue specimens. Scale bars: 50 μm. (G) The correlation between KLF12 and PD‐L1 was shown and evaluated by Pearson's correlation test (*p* < 0.0001, *r* = 0.423) (*n* = 33). *n*, number of cases. –, 0 < expression rate <1%, +, 1 < expression rate < 25%, ++, 25% < expression rate < 50%, +++, expression rate > 50%. (H) PFS and OS of 110 NSCLC patients based on tissue microarray (related to Fig. S1C,D) were estimated by Kaplan–Meier survival curves. The rate of positively stained tissue chip less than 50% was classified into low expression category, while positively stained tissue chip more than 50% high expression category. The difference of survival curves was conducted via Gehan–Breslow–Wilcoxon test. HR: Hazard Ratio. Data were shown as mean ± SD. Statistical significance of differences between two groups was determined by Student’s *t*‐test. *, *P* < 0.05; **, *P* < 0.01; ***, *P* < 0.001; n.s., *P* > 0.05.

The levels of PD‐L1 protein in NSCLC exhibit considerable heterogeneity [20]; however, the existing mechanisms of PD‐L1 expression cannot be fully explained. To characterize the transcriptome difference between PD‐L1‐expressing and PD‐L1‐nonexpressing cells, NCI‐H460 cells, which express relatively higher levels of PD‐L1, were labeled with a PE‐conjugated PD‐L1 antibody and sorted. According to the relative fluorescence intensity (RFI), PD‐L1 expression (RFI was approximately 10^4^) and nonexpression cells (RFI was approximately 10^2^) were identified (Fig. 1A), and the transcriptome of these cells was analyzed by whole‐genome gene chip (Fig. S1A). Among the 400 protein‐coding genes that were upregulated more than twofold in PD‐L1‐expressing cells, four transcription factors upregulated more than fourfold, including *KLF12*, *TCF7*, *ZNF703*, and *ZNF623*, were chosen for further confirmation (Fig. 1B). The data showed that KLF12 was significantly upregulated (3.55‐fold), whereas no significant difference was observed for the other three transcription factors. (Fig. 1C). A positive correlation between KLF12 and PD‐L1 proteins was also found in cells sorted from NCI‐H460 and NCI‐H1299 cells (Fig. 1D,E). Gene Expression Profiling Interactive Analysis (GEPIA) also showed a positive correlation between *PD‐L1* and *KLF12* (Fig. S1B).

To further evaluate the correlation between KLF12 and PD‐L1 expression, we analyzed their correlation in NSCLC patient tissue specimens using IHC (Fig. 1F and Table S4). A positive correlation between KLF12 and PD‐L1 expression in NSCLC patient tissue specimens was determined with Pearson's chi‐squared *t*est (*r* = 0.423) (Fig. 1G). The same result was also observed in NSCLC tissue microarray (*r* = 0.5238) (Fig. S1C,D). Furthermore, we analyzed the correlation between KLF12 or PD‐L1 expression and progression‐free survival (PFS) and overall survival (OS). As expected [21], shorter PFS and OS were observed in lung cancer patients with high expression of PD‐L1 (Fig. 1H and Table S5), as well as high expression of KLF12. Interestingly, we found that NSCLC patients with low expression of KLF12 had more proportion of TNM stage I, while high percentage of stage II‐IV were investigated in high KLF12 expression (Fig. S1E). This data suggested that KLF12 expression in lung cancer tissues may be related to the TNM stage in patients. The impact of KLF12 expression on survival rates was further evaluated with PrognoScan. Notably, high KLF12 expression significantly impacted prognosis (Fig. S1F). These data all indicated a positive relationship between KLF12 and PD‐L1 expression.

### 3.2 KLF12 binds to the *PD‐L1* promoter and promotes *PD‐L1* expression

**Fig. 2:**
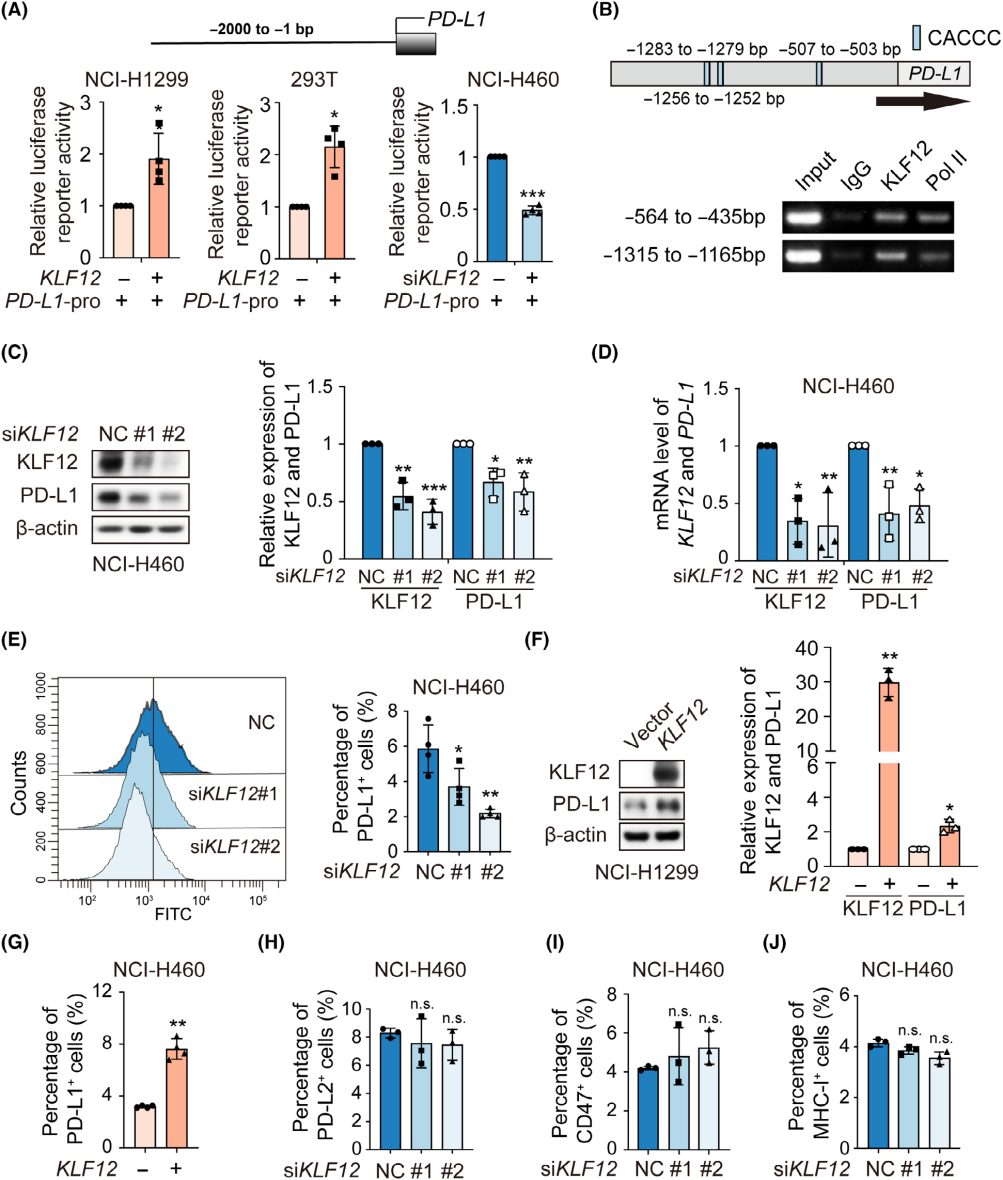
KLF12 bound to *PD‐L1* promoter and upregulated *PD‐L1* transcription. (A) NCI‐H1299 cells and 293T cells were transfected with *PD‐L1* promoter luciferase reporter plasmids and *KLF12*‐HA for 24 h. Simultaneously, NCI‐H460 cells were transfected with *PD‐L1* promoter luciferase reporter plasmids and *KLF12* siRNA for 24 h. Then, their relative luciferase activity was detected using the dual‐luciferase reporter assay. The experiments were performed in quadruplicate. (B) The *PD‐L1* promoter region containing CACCC‐box was retrieved from NCBI database (up). And the binding of KLF12 to *PD‐L1* promoter was determined by ChIP assay (bottom). The anti‐rabbit IgG and anti‐RNA polymerase II (Pol II) antibodies were considered as negative and positive control. The experiments were performed in duplicate. (C) The expression of KLF12 and PD‐L1 in NCI‐H460 was measured by western blot after *KLF12* silencing. Relative change of KLF12 or PD‐L1 protein was determined by densitometric analysis. The experiments were performed in triplicate. (D) The mRNA levels of *KLF12* and *PD‐L1* genes in NCI‐H460 were detected by q‐RT‐PCR after *KLF12* silencing. The experiments were performed in triplicate. (E) Cell‐surface PD‐L1 expression in NCI‐H460 was detected by flow cytometry after *KLF12* silencing. The experiments were performed in quadruplicate. (F) The expression of KLF12 and PD‐L1 was detected by western blot when NCI‐H1299 was transfected with *KLF12*‐HA, and the relative change of KLF12 or PD‐L1 expression was determined by densitometric analysis. The experiments were performed in triplicate. (G) Cell‐surface PD‐L1 expression in NCI‐H460 with transient *KLF12* overexpression was detected by flow cytometry. The experiments were performed in quadruplicate. (H–J) Cell‐surface PD‐L2 (H), CD47 (I), and MHC‐I (J) expression in NCI‐H460 with transient *KLF12* knockdown was detected by flow cytometry. The experiments were performed in triplicate. Data were presented as mean ± SD. Statistical analysis of the data was performed by Student's *t*‐test (two groups) and one‐way ANOVA with Dunnett's *post hoc* test (more than two groups). *, *P* < 0.05; **, *P* < 0.01; ***, *P* < 0.001; n.s., *P* > 0.05.

As KLF12 is a transcription factor of the KLF family, we next estimated whether KLF12 could transcriptionally regulate *PD‐L1* expression. We constructed luciferase reporter plasmids bearing the −2000 bp region of the *PD‐L1* promoter. *PD‐L1* promoter activity was enhanced after *KLF12* overexpression via dual‐luciferase reporter assay but decreased by *KLF12* knockdown. (Fig. 2A). Analysis of KLF12 binding site data showed three putative KLF12 binding sites on the *PD‐L1* promoter at −507 bp, −1256 bp, and −1283 bp relative to the transcription start site. ChIP results showed that KLF12 bound to the “CACCC‐box” region of the *PD‐L1* promoter (Fig. 2B). In addition, both the protein and mRNA levels of PD‐L1 were decreased after *KLF12* silencing in NCI‐H460 (Fig. 2C,D) and NCI‐H1299 cells (Fig. S2A,B). Similar effects by *KLF12* knockdown were observed in an EGFR‐mutant NSCLC cell line, NCI‐H1975 (Fig. S2C). Moreover, flow cytometry demonstrated that *KLF12* knockdown attenuated the expression of cell membrane surface PD‐L1 in H460 and H1299 (Fig. 2E and Fig. S2D). Consistent with this finding, increased expression of PD‐L1 was found with *KLF12* overexpression (Fig. 2F and Fig. S2E). Additionally, cell‐surface PD‐L1 expression was upregulated with *KLF12* overexpression (Fig. 2G). In parallel, we further tested the effect of KLF12 on the expression of additional cell surface immunosuppressive molecules PD‐L2, CD47, and MHC I. The results showed that the cell surface expression of PD‐L2, CD47, and MHC I was not significantly affected by *KLF12* intervention (Fig. 2H–J).

Next, we examined whether the stability of PD‐L1 proteins was affected by *KLF12* silencing. The results showed that the half‐life of PD‐L1 was unaffected by *KLF12* knockdown (Fig. S2F). Moreover, neither mRNA nor protein levels of KLF12 were changed with *PD‐L1* siRNA (Fig. S2G,H). Taken together, these data suggest that KLF12 serves as a transcription factor to directly regulate *PD‐L1* expression.

### 3.3 STAT1/3‐mediated *PD‐L1* expression is disrupted by KLF12 knockdown

**Fig. 3:**
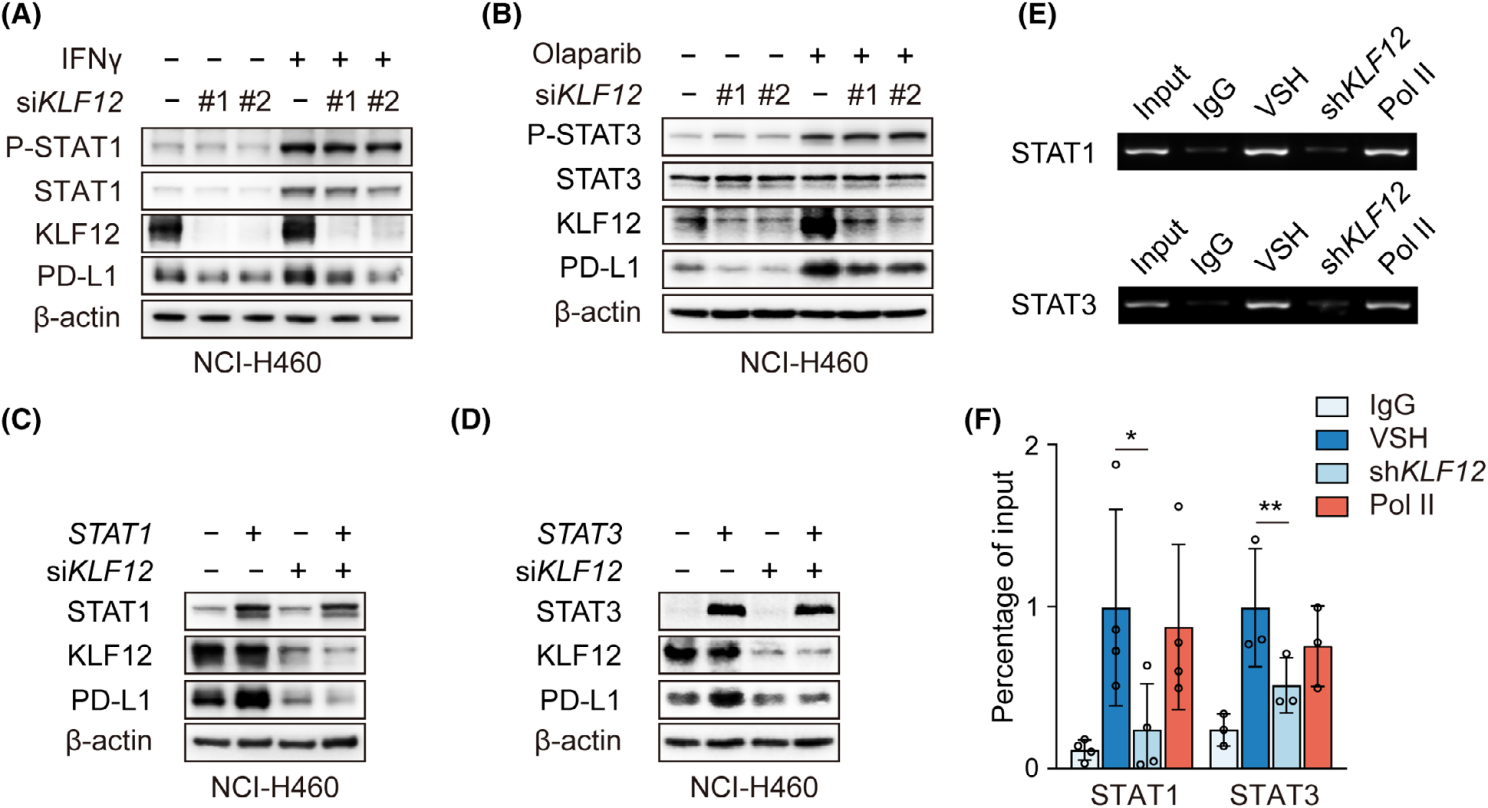
*KLF12* knockdown attenuated signal transducer and activator of transcription (STAT) 1/3‐mediated the transcription of *PD‐L1*. (A) P‐STAT1, STAT1, KLF12, and PD‐L1 proteins in NCI‐H460 cells with transient *KLF12* knockdown and interferon gamma (IFNγ) (10 ng·μL^−1^) treatment were evaluated by western blot. The experiments were performed in triplicate. (B) P‐STAT3, STAT3, KLF12, and PD‐L1 proteins in NCI‐H460 cells with transient *KLF12* knockdown and Olaparib (10 μm) treatment were evaluated by western blot. The experiments were performed in duplicate. (C, D) NCI‐H460 cells transfected with transient *KLF12* knockdown were treated with *STAT1* or *STAT3* overexpression. The expression of STAT1, KLF12, and PD‐L1 proteins (C) and STAT3, KLF12, and PD‐L1 proteins (D) was detected. The experiments were performed in duplicate. (E, F) Binding of STAT1 and STAT3 to the *PD‐L1* promoter after shRNA targeting *KLF12* (sh*KLF12*). Sheared chromatin from vector control (VSH) and sh*KLF12* NCI‐H460 was immunoprecipitated with STAT1 and STAT3 polyclonal antibodies. IgG antibody as the negative control and RNA polymerase II (Pol II) antibody as the positive control. PCR (E) and qPCR (F) were performed using primer pairs specific for promoter of the *PD‐L1* gene. ChIP data were normalized to Input. The experiments were repeated at least for three times. Data were shown as mean ± SD. Statistical analysis of the data was performed by Student's *t*‐test. *, *P* < 0.05; **, *P* < 0.01.

Considering that interferon gamma (IFNγ), PARP1 inhibitors (PARPis), and their downstream JAK/STAT signaling pathways have been reported to stimulate *PD‐L1* expression [22, 23], we also explored whether this was also the case with *KLF12* knockdown and decreased PD‐L1 expression. Interestingly, knockdown of *KLF12* inhibited the IFNγ‐induced upregulation of PD‐L1 protein without affecting the phosphorylation of downstream STAT1 (Fig. 3A). A similar result was observed in the Olaparib‐induced PD‐L1 model (Fig. 3B). Consistently, *STAT1* and *STAT3* overexpression upregulated PD‐L1 expression, whereas *KLF12* knockdown attenuated STAT1‐ and STAT3‐induced PD‐L1 expression (Fig. 3C,D). STAT1 [24] and STAT3 [25] activate *PD‐L1* transcription by directly binding to the promoter region. To further delineate whether KLF12 interfered with the binding of STAT1/3 to *PD‐L1* promoter regions, we performed a ChIP assay to measure the binding of STAT1 and STAT3 to *PD‐L1* promoter regions. The blunted binding of STAT1 and STAT3 to the *PD‐L1* promoter was observed after *KLF12* knockdown by PCR (Fig. 3E) and qPCR (Fig. 3F). These data suggest that *KLF12* knockdown inhibited STAT1/3‐mediated PD‐L1 expression.

### 3.4 KLF12 mediates P300‐induced histone H3 acetylation

**Fig. 4:**
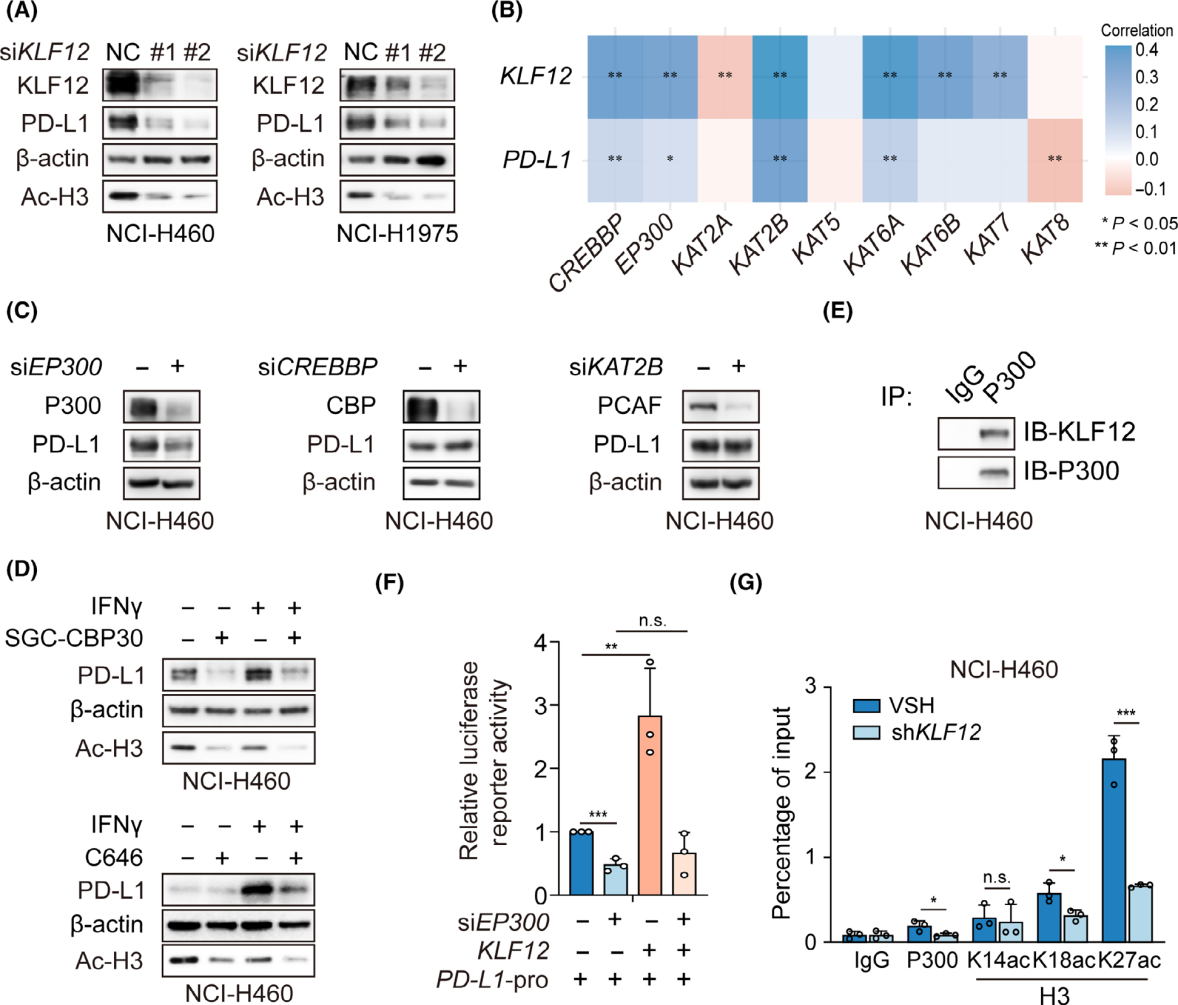
KLF12 recruited P300 to acetylate histone H3 of *PD‐L1* promoter region. (A) KLF12, PD‐L1, and Ac‐H3 proteins in NCI‐H460 cells with transient *KLF12* knockdown were evaluated by western blot. The experiments were performed in duplicate. (B) The correlation of 9 lysine acetyltransferases, KLF12, and PD‐L1 in TCGA. The correlation was performed by Student's *t*‐test (*n* = 516). (C) The expression of PD‐L1 in NCI‐H460 cells transfected with transient *EP300* (encoding P300), *CREBBP* (encoding CBP), or *KAT2B* (encoding PCAF) knockdown was detected. The experiments were performed in triplicate. (D) The expression of PD‐L1 in NCI‐H460 cells treated with P300 inhibitors SGC‐CBP30 (10 μm) and C646 (10 μm) in the presence of IFNγ (10 ng·μL^−1^) was detected. The experiments were performed in duplicate. (E) Coimmunoprecipitation (co‐IP) assay was conducted with P300 antibody in NCI‐H460 cells, which was followed by immunoblotting with the KLF12 antibody. The experiments were performed in duplicate. (F) *PD‐L1* promoter activity in NCI‐H460 cells transfected with *EP300* siRNA and *KLF12* overexpression. The experiments were performed in triplicate. (G) ChIP‐qPCR of *PD‐L1* promoter region was conducted with IgG, P300, H3K14ac, H3K18ac, and H3K27ac antibody in NCI‐H460 cells with vector control (VSH) or shRNA‐mediated knockdown of *KLF12* (sh*KLF12*). The experiments were performed in triplicate. Data were shown as mean ± SD. Statistical analysis of the data was performed by Student's *t*‐test. *, *P* < 0.05; **, *P* < 0.01; ***, *P* < 0.001; n.s., *P* > 0.05.

Generally, histone acetylation enhances the binding of transcription factors to the promoter region of target genes [26, 27]. As a previous report on KLFs showed the regulation of histone H3 acetylation [28, 29, 30], we next investigated whether KLF12 influences histone H3 acetylation of PD‐L1. We found that the knockdown of *KLF12* reduced the Ac‐H3 (Fig. 4A and Fig. S3A). To determine the lysine acetyltransferase(s) involved, we assessed the correlation of lysine acetyltransferase(s) with *KLF12* and *PD‐L1* in The Cancer Genome Atlas (TCGA). As shown in Fig. 4B, *CREBBP*, *EP300*, *KAT2B*, and *KAT6A* correlated positively with *KLF12* and *PD‐L1* expression. Notably, *CREBBP*, *EP300*, and *KAT2B* have been reported to be recruited by KLF family proteins. Thus, we knocked down *CREBBP*, *EP300*, and *KAT2B* by siRNA and found that *EP300* knockdown greatly reduced PD‐L1 expression (Fig. 4C and Fig. S3B). SGC‐CBP30 and C646, selective inhibitors of the P300, also attenuated PD‐L1 expression and histone H3 acetylation (Fig. 4D). These data suggest that P300 is involved in PD‐L1 expression. Coimmunoprecipitation (co‐IP) analysis identified that P300 interacted with KLF12 (Fig. 4E). In addition, *KLF12* overexpression enhanced *PD‐L1* transcription, and this effect was abrogated when *EP300* was knocked down (Fig. 4F). Furthermore, ChIP assays showed that P300 binding to the *PD‐L1* promoter region was reduced after *KLF12* knockdown. Specifically, among P300 histone lysine acetylation sites, H3 lysine 18 (H3K18) and H3K27 acetylation, but not H3K14 acetylation, was significantly reduced after *KLF12* knockdown (Fig. 4G and Fig. S3C). These data indicate that KLF12 promotes p300 recruitment, which leads to improved acetylation of H3K18 and H3K27 and subsequently *PD‐L1* transcription.

### 3.5 KLF12 deficiency leads to an enhanced antitumor effect

**Fig. 5:**
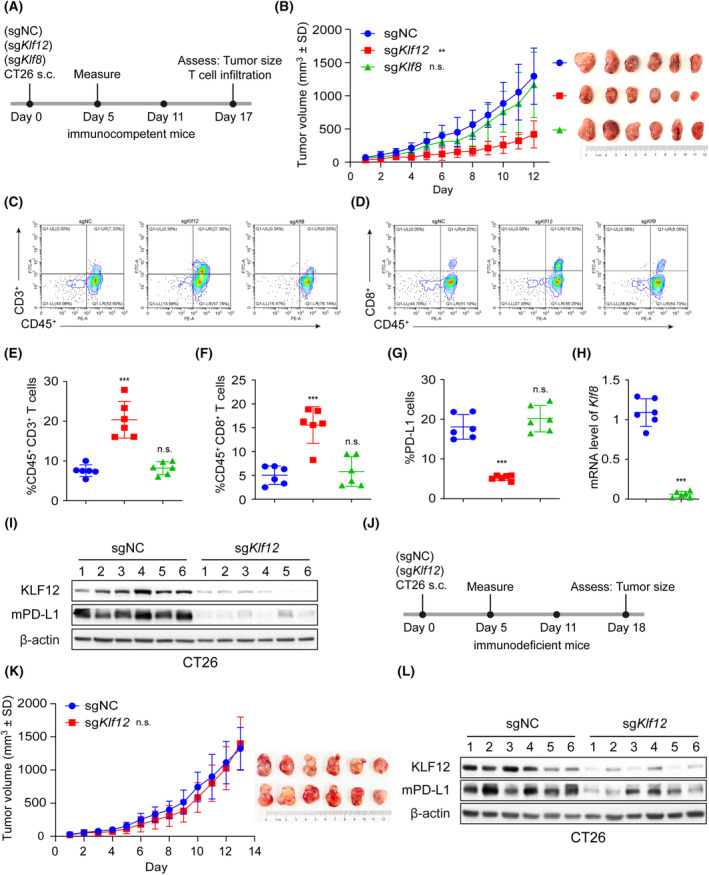
*Klf12* knockout inhibited PD‐L1 expression and enhanced T cell activity *in vivo*. (A) Schematic diagram of the experimental procedure. The immunocompetent BALB/c mice were inoculated with sgNC, sg*KLF8*, and sg*KLF12* CT26 cells (*n* = 6/group). s.c.: subcutaneous injection. (B) The tumor growth curves and tumor images of BALB/c mice bearing the indicated KO CT26 cells (*n* = 6/group). (C, D) Representative images of tumor‐infiltrating CD45^+^CD3^+^ T cells (C) and CD45^+^CD8^+^ T cells (D) in sgNC group (*n* = 6), sg*KLF8* group (*n* = 6), and sg*KLF12* group (*n* = 6). (E–G) The tumor‐infiltrating CD45^+^CD3^+^ T cells (E), CD45^+^CD8^+^ T cells (F), and tumor PD‐L1 levels (G) in sgNC group (*n* = 6), sg*KLF8* group (*n* = 6), and sg*KLF12* group (*n* = 6) were analyzed by flow cytometry. (H, I) The murine *Klf8* mRNA (H), KLF12, and PD‐L1 (mPD‐L1) proteins (I) in mouse tumor tissues of each group were respectively measured by q‐RT‐PCR and western blot (*n* = 6/group). (J) Schematic diagram of the experimental procedure. The immunodeficient mice were inoculated with sgNC and sg*KLF12* CT26 cells (*n* = 6/group). (K) The tumor growth curves and tumor images of immunodeficient mice bearing the indicated KO CT26 cells (*n* = 6). (L) The KLF12 and mPD‐L1 proteins in mouse tumor tissues were measured by western blot (*n* = 6/group). Data were presented as mean ± SD. Statistical analysis of the data was performed by Student's *t*‐test (two groups) and one‐way ANOVA with Dunnett's *post hoc* test (more than two groups). **, *P* < 0.01; ***, *P* < 0.001; n.s., *P* > 0.05.

It has been reported that KLF proteins are highly conserved among mammals from human to mouse [3, 31]. Mouse KLF12 contains 402 amino acids and shares 98% identity with human KLF12. The DNA sequence of murine *Pd‐l1* also contained “CACCC” motif (Fig. S4A). Next, we investigated whether KLF12 regulates T‐cell–mediated antitumor immunity via PD‐L1. We generated CRISPR/Cas9‐mediated knockout (KO) of negative control (NC) and *Klf12* in mouse colon cancer CT26 cells, and we also generated KO of *Klf8* as a control. As shown in Fig. S4B, *Klf12* deletion significantly inhibited H3 acetylation and PD‐L1 levels in CT26 cells. Then, the *Klf12*‐KO CT26 cells were subcutaneously injected into BALB/c mice. The tumor growth curve of each mouse was monitored every day, and T cells infiltrating into tumors were harvested after 12 days (Fig. 5A). *Klf12* deletion significantly reduced tumor burden compared with NC, whereas no significant difference was found in the NC and *Klf8* KO groups (Fig. 5B). In addition, *Klf12* deletion instead of *Klf8* deletion increased the percentage of CD3^+^ T cells and CD8^+^ T cells in the tumors compared with NC (Fig. 5C–F). Consistent with our previous results, PD‐L1‐positive cells in tumors were significantly suppressed by *Klf12* deficiency (Fig. 5G). Moreover, the depleted levels of *Klf8* and *Klf12* and the expression of PD‐L1 were further determined by q‐RT–PCR and western blot (Fig. 5H,I).

To determine whether the KLF12 effect is dependent upon the immune system, we next analyzed the contribution of *Klf12* deficiency to tumor growth in immunodeficient mice. Immunodeficient BALB/c nude mice were injected subcutaneously with NC and *Klf12* KO CT26 cells (Fig. 5J). We observed that *Klf12* KO tumors exhibited similar tumor growth as control tumors, while *Klf12* KO tumors maintained lower KLF12 and PD‐L1 expression compared with control tumors (Fig. 5K,L). These findings implied that *KLF12* deficiency promoted T‐cell–mediated antitumor immunity and consequent antitumor effects, and the immune system was necessary for antitumor effects via KLF12 deficiency.

## 4 Discussion

Here, we showed that KLF12 is positively correlated with PD‐L1 expression in NSCLC patient tumor tissues. KLF12 was identified as the transcription factor of PD‐L1 that promotes *PD‐L1* transcription. Further mechanistic studies revealed that *KLF12* knockdown reduces P300 recruitment to the *PD‐L1* promoter, thereby intervening in the binding of STAT1 and STAT3 at the *PD‐L1* promoter. Furthermore, *KLF12* knockdown promoted antitumor effects by enhancing CD8^+^ T‐cell infiltration. Therefore, we identified a novel transcription factor for PD‐L1 in NSCLC and revealed a molecular mechanism that regulates *PD‐L1* transcription. We also revealed a potential therapeutic strategy for NSCLC by inhibiting KLF12.

KLF12, initially identified as a transcriptional repressor, represses the transcription of AP‐2α [4]. KLF12 contains a conserved PVDLS region at the N terminus, which can bind directly to the corepressor C‐terminal‐binding protein (CtBP) [32, 33] to inhibit the expression of target genes, such as Nur77 and FOXO1 [34, 35]. However, KLF12 is reported to function as a transcriptional activator of downstream genes, including *EGR1* and *SLC14A1* [6, 36]. Similar to KLF12, KLF8 also plays various roles in transcriptional repression or activation by recruiting corepressors or coactivators [30, 37]. Additionally, KLF4 can activate and repress the transcription of genes depending not only on the contents of target genes and their interacting partner proteins [38]. Thus, KLF12 can either repress or activate the transcription of target genes, depending on the recruited cofactors. Our findings revealed that KLF12 served as a transcriptional activator of PD‐L1 (Fig. 2A). Currently, our research mainly focuses on NSCLC, in which the pathological background of KLF12 plays a significant role in regulating *PD‐L1*, is still a problem to be solved. Additionally, our findings suggested that KLF12 expression might be influenced by IFNγ and PARPi (Fig. 2A,B); thus, the regulation of KLF12 is worth further exploration.

Histones are subjected to a variety of posttranslational modifications (PTMs) [39], among which the most extensively studied is histone acetylation. Generally, acetylation of histones relaxes chromatin structure by neutralizing the positive charge of chromatin, activating gene transcription [40, 41]. Recent investigations have emphasized that KLF4 increases the levels of acetylated H4 at the promoter of intestinal alkaline phosphatase (*IAP*) by interacting with the P300, thereby increasing the expression of IAP [42]. The interaction between KLF4 and P300 acetylates histone H3 and activates transcription at the gene promoter of *VSMCs* [28]. KLF8 recruits P300 and PCAF to target gene promoters followed by promotion of histone acetylation and transcriptional activation [30]. Therefore, we first investigated potential lysine acetyltransferase(s) and found that P300 might be involved in the transcriptional regulation of KLF12. Whether other epigenetic regulatory factors contribute to KLF12 regulation of PD‐L1 is worthy of further investigation.

Based on accumulated clinical evidence, PD‐L1 expression is now widely accepted as a biomarker for anti‐PD‐1/PD‐L1 immunotherapy in NSCLC [43]. Patients with high expression of PD‐L1 have better efficacy than those with low or no expression. However, the limitations of IHC detection of PD‐L1 expression have led to conflicting results [44]. Due to different scoring systems and the diversity of antibodies, PD‐L1 detection by IHC differs between clinical analyses. Furthermore, temporal and spatial heterogeneity of PD‐L1 expression leads to discordance between biopsy specimens and resected tissue [45]. Our study showed a positive correlation between KLF12 and PD‐L1 expression in clinical patient tumor tissues. Additionally, patients with low KLF12 expression had longer PFS than those with high KLF12 expression. Therefore, the levels of KLF12 may be used to assess PD‐L1 expression and anti‐PD‐1/PD‐L1 efficacy.

## 5 Conclusions

Taken together, our study revealed that KLF12 serves as a transcription factor to enhance PD‐L1 transcription by recruiting the lysine acetyltransferase the P300. Our findings not only enrich the mechanism of PD‐L1 regulation in NSCLC and identify KLF12 as a novel transcription factor for PD‐L1 but also provide a basis for targeting KLF12 for NSCLC immunotherapy.

## Conflict of interest

The authors declare no conflict of interest.

## Author contributions

XP and WZ contributed to the design, conducted experiments of the work, and wrote the manuscript; LW, HG, MZ, HW, and QH contributed to the acquisition of the work and revised the article; QW contributed to the animal study and provided project supervision. LD and BY contributed to the conception of the work and provided project supervision.

## Supporting information


**Fig. S1.** Positive correlation between KLF12 and PD‐L1 in NSCLC.
**Fig. S2.** KLF12 transcriptionally regulated *PD‐L1* expression.
**Fig. S3.** KLF12 recruited P300 to *PD‐L1* promoter region by promoting P300‐mediated H3 acetylation.
**Fig. S4.** *Klf12* knockout inhibited PD‐L1 expression.
**Table S1.** Different genes in gene chip analysis.
**Table S2.** siRNA Sequence.
**Table S3.** Primers for q‐RT‐PCR.
**Table S4.** Clinicopathologic characteristics of 33 clinical lung cancer patient tissues.
**Table S5.** Clinicopathologic characteristics of lung cancer tissues microarray cohorts.

## Data Availability

The data supporting in manuscript are available in the article. Source data for all figures and supplementary information have been provided with this paper.
