# Supplementary material for: KLF12 transcriptionally regulates PD‐L1 expression in non‐small cell lung cancer
Source: Mol Oncol. 2023 Sep 2;17(12):2659–74. doi: 10.1002/1878-0261.13512 (PMC10701771; doi:10.1002/1878-0261.13512)
Supplement: Supplementary file 1 — Fig. S1. Positive correlation between KLF12 and PD‐L1 in NSCLC. Fig. S2. KLF12 transcriptionally regulated PD‐L1 expression. Fig. S3. KLF12 recruited P300 to PD‐L1 promoter region by promoting P300‐mediated H3 acetylation. Fig. S4. Klf12 knockout inhibited PD‐L1 expression. Table S1. Different genes in gene chip analysis. Table S2. siRNA Sequence. Table S3. Primers for q‐RT‐PCR. Table S4. Clinicopathologic characteristics of 33 clinical lung cancer patient tissues. Table S5. Clinicopathologic characteristics of lung cancer tissues microarray cohorts. [file MOL2-17-2659-s001.docx]

**Supplementary Figures**


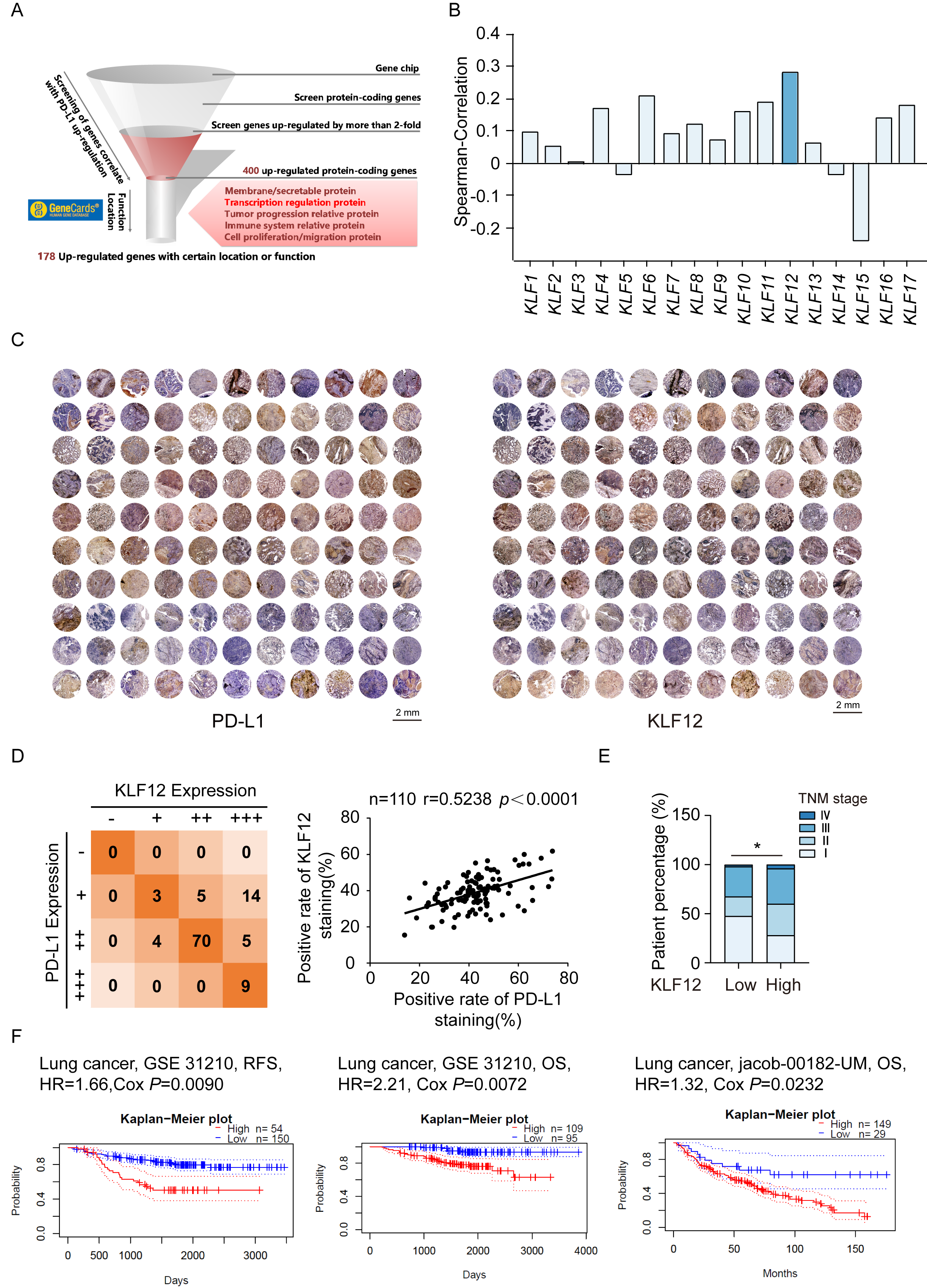


**Figure S1 The positive correlation between KLF12 and PD-L1 in NSCLC. A** The sorted NCI-H460 cells with high or low PD-L1 membrane expression were sent for gene chip study. The protein-coding genes which up-regulated by more than 2-fold were screened and investigated by Genecards (n=2). **B** The correlation of *KLF* family expression and *PD-L1* expression in Gene Expression Profiling Interactive Analysis (GEPIA) (n=17). **C** IHC staining of PD-L1 and KLF12 in non-small cell lung cancer (NSCLC) tissue microarray (n=110/group). Scale bars: 2 μm. **D** The correlation of KLF12 and PD-L1 in NSCLC tissue microarray was showed and evaluated by Pearson correlation test (*p* < 0.0001, r = 0.5238) (n=110/group). -, 0 < expression rate < 1%, +, 1 < expression rate < 25%, ++, 25% < expression rate < 50%, +++, expression rate > 50%. **E** Different KLF12 level of NSCLC patient tumor tissues stratified by different TNM stage. Assessment data of the TNM stage among NSCLC patients were performed using Fisher’s exact *t*est (Low KLF12 expression, n=102; High KLF12 expression, n=41). **F** The Kaplan-Meier survival curves comparing KLF12 expression in NSCLC in the PrognoScan. *, *p* < 0.05.


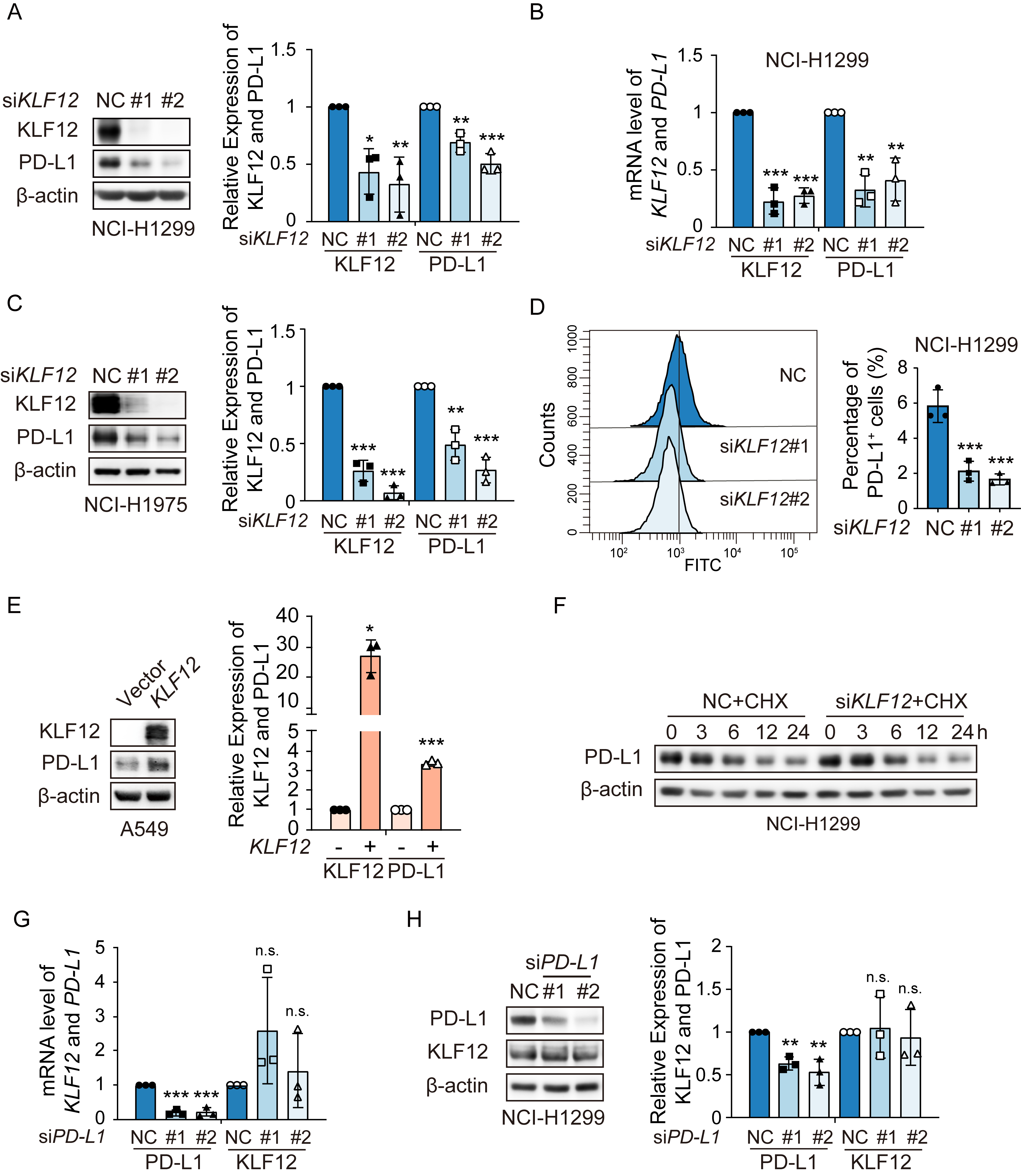


**Figure S2 KLF12 transcriptionally regulated *PD-L1* expression. A, B** The expression of KLF12 and PD-L1 in NCI-H1299 (A) and NCI-H1975 (B) cells with *KLF12* knockdown was measured by western blot. Relative change of KLF12 or PD-L1 protein was determined by densitometric analysis. The experiments were performed in triplicate. **C** The mRNA level of *KLF12* and *PD-L1* in NCI-H1299 cells with *KLF12* knockdown were detected by q-RT-PCR. The experiments were performed in triplicate. **D** Cell-surface PD-L1 expression in NCI-H1299 cells was detected by flow cytometry after *KLF12* silencing. The experiments were performed in triplicate. **E** The expression of KLF12 and PD-L1 was detected by western blot when A549 cells were transfected with *KLF12*-HA, and the relative change of KLF12 or PD-L1 expression was determined by densitometric analysis. The experiments were performed in triplicate. **F** NCI-H1299 cells were transfected with *KLF12* siRNA for 12 hours, and then treated with 10 μg/mL cycloheximide (CHX) for different time (0, 3, 6, 12, and 24 hours). Protein expression of PD-L1 was detected by western blot. The experiments were performed in triplicate. **G** The mRNA levels of *KLF12* and *PD-L1* in NCI-H1299 cells with *PD-L1* knockdown were detected by q-RT-PCR. The experiments were performed in triplicate. **H** The expression of KLF12 and PD-L1 in NCI-H1299 cells with *PD-L1* knockdown was detected by western blot, and the relative change of KLF12 or PD-L1 expression was determined by densitometric analysis. The experiments were performed in triplicate. Data were presented as the mean ± SD. Data were presented as mean ± SD. Statistical analysis of the data were performed by Student’s *t*-test (two groups) and one-way ANOVA with Dunnett’s post hoc test (more than two groups). *, *p* < 0.05; **, *p* < 0.01; ***, *p* < 0.001; n.s., *p* > 0.05.


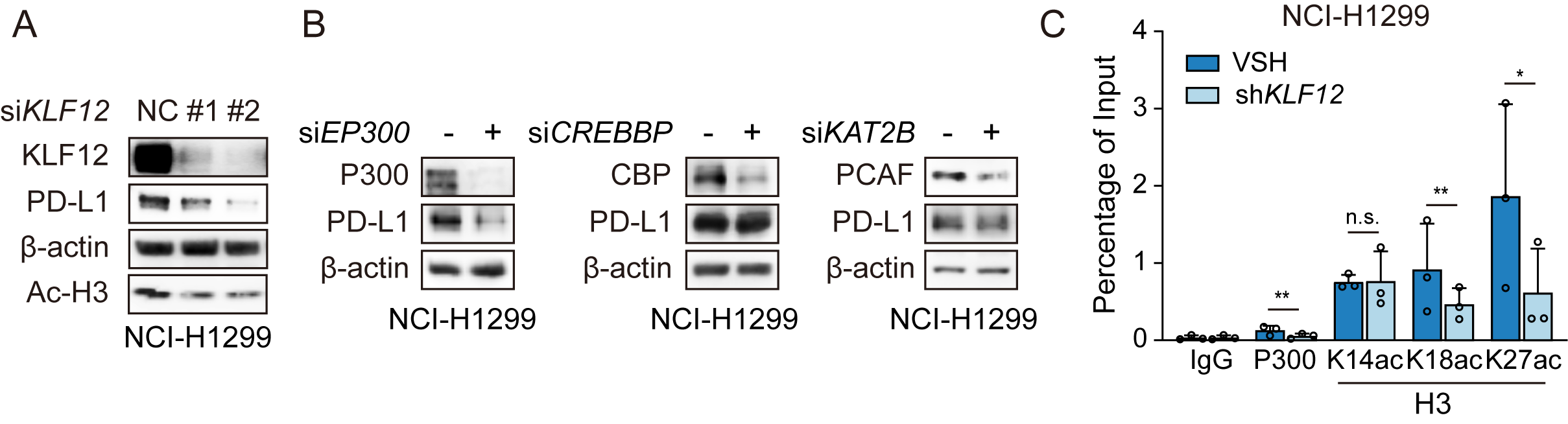


**Figure S3** **KLF12 recruited P300 to *PD-L1* promoter region by promoting P300-mediated H3 acetylation. A** KLF12, PD-L1 and acetylation of histone H3 (Ac-H3) levels in NCI-H1299 cells with *KLF12* silencing were evaluated by western blot. The experiments were performed in duplicate. **B** The expression of PD-L1 in NCI-H1299 cells transfected with transient *EP300* (encoding P300), *CREBBP (encoding CBP)*, or *KAT2B* (encoding PCAF) knockdown was detected. The experiments were performed in duplicate. **C** ChIP-qPCR of *PD-L1* promoter region was conducted with IgG, P300, H3K14ac (H3 lysine 14 acetylation), H3K18ac, and H3K27ac antibody in vector control (VSH) or shRNA-mediated knockdown of *KLF12* (sh*KLF12*) in NCI-H1299 cells. The experiments were performed in triplicate. IgG antibody as the negative control and ChIP data were normalized to Input. Data were shown as mean ± SD. Student’s *t*-test was used to determine Statistical differences. *, *p* < 0.05; **, *p* < 0.01; n.s., *p* > 0.05.


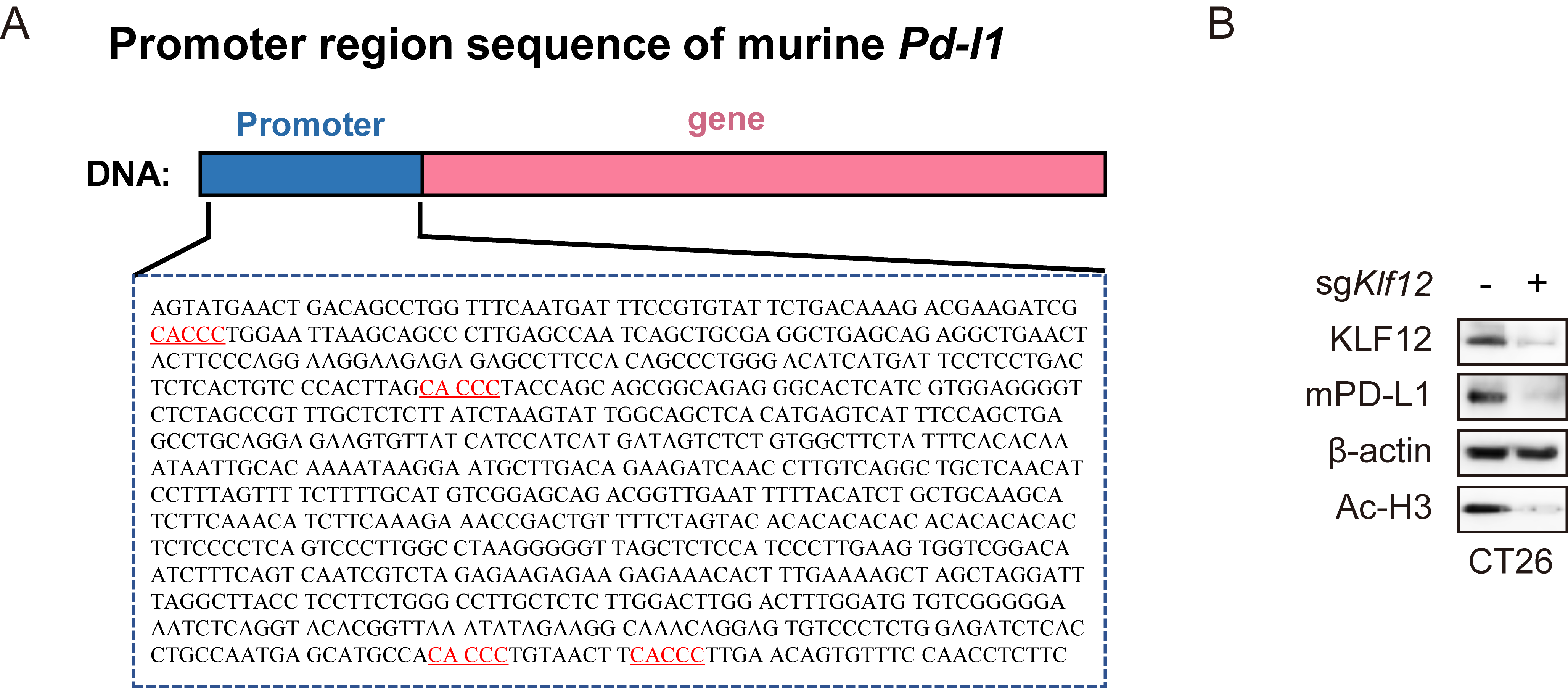


**Figure S4 *Klf12* knockout inhibited PD-L1 expression. A** The predicted hits of the KLF12 signature motif in murine *Pd-l1* promoter region were assayed. **B** The expression of KLF12, murine PD-L1, and acetylation of histone H3 (Ac-H3) in sg*Klf12* CT26 cells was detected. The data were the results of one experiment.

**Supplementary Tables**

Table S1 The different genes in gene chip analysis

| Classification (number) | Gene names |
| --- | --- |
| Membrane/secretable protein (96) | *TACA1, THRB, PLP1, LYPD1, SUSD2, PAPPA, PCDH15, SEMA5A, SCN1A, SPRR2A, VCPIP1, LGI1, NLGN1, FAT2, GRHL2, COL13A1, ADTRP, NRP1, AGTR2, ADAMTS16, GCSAM, KCNMA1, BFSP2, FBN2, CCL21, CDHR3, PTPRN2, RASSF3, HCRTR2, HAS3, MPP4, IGFBP7, LTBP1, PRSS38, ANXA3, GJB7, KCNG4, EPHA5, IRS1, PLAU, JPH2, HS3ST3B1, ADCY2, RGS2, ADAM28, ATP9B, CD55, LILRA6, CLCN6, THBD, ABCA3, CEACAM1, GPR85, CPAMD8, GPR65, LAMA43, PTPRG, SEMA6C, DLCK1, IGSF3, PTP4A3, TMEM154, NTSR1, LYPD5, GPRC5D, PIGZ, ANTXR2, IL2RG, PRPH2, PDGFRL, PLCH2, EHD2, TAS1R3, CDCP1, NT5E, ITGBL1, PIK3R1, CACNB4, KCNG3, PLXNA2, KIR2DL2, PTPRF, TNFRSF6B, TSPAN5, TMEM56, B3GNT3, C14ORF180, INSIG2, MTMR1, GPR160, PIGQ, EHBP1, PSD4* |
| Transcription regulation protein (34) | *KLF12, TCF7, ZNF703, ZNF623, ZNF449, ZMYM2, PRDM8, FOXN3, MED1, ZNF132, ZNF776, ZNF468, PRMD2, YAF2, ZBED6, DMRT2, GLI2, MAMLD1, ARID5B, PRDM16, EYA4, ENC1, TRPS1, ZNF25, CEBPA, CITED4, SOX7, ZNF81, PHF10, FOXP1, NKX2-4, KDM3A, ZSCAN16, HOXA2* |
| Tumor progression relative protein (17) | *RNF6, GKN1, DLC1, ARMCX1, CDC14B, NBPF6, PRUNE2, TAL1, IL24, SMAD2, PKN, NEDD9, ARMC3, HID1, HYAL1, GKN2, MDM2* |
| Immune system relative protein (12) | *LGALS13, CD4, DEFA6, TNFAIP8L2, LYZL2, PTX3, DEFB1, SH2D2A, TGFB2, OAS3, CYFIP2, THEMIS2* |
| Cell proliferation/ migration protein (19) | *CA9, SCGB1D4, GDF2, DACT1, CASP8AP2, NEK1, CPNE4, REM1, SRCIN1, FRK, MEIOB, LAMB1, PMEPA1, CYR61, LSM11, RIF1, CSH2, CDK6, LAMA1* |

Table S2 siRNA Sequence

| Gene name | | Primer Sequence (5’→3’) |
| --- | --- | --- |
| Negative  Control | Sense | UUCUCCGAACGUGUCACGUTT |
|  | Anti-sense | ACGUGACACGUUCGGAGAATT |
| si*PD-L1* #1 | Sense  Anti-sense | GAGGAAGACCUGAAGGUUCAGCAUA  UAUGCUGAACCUUCAGGUCUUCCUC |
| si*PD-L1* #2 | Sense  Anti-sense | CCUACUGGCAUUUGCUGAACGCAUU  AAUGCGUUCAGCAAAUGCCAGUAGG |
| si*KLF12* #1 | Sense | CCUACUGGCAUUUGCUGAACGCAUU |
|  | Anti-sense | AAUGCGUUCAGCAAAUGCCAGUAGG |
| si*KLF12* #2 | Sense | UGAUACACAUUUGGAGGAGACGUAA |
|  | Anti-sense | UUACGUCUCCUCCAAAUGUGUAUCA |
| si*EP300* | Sense | GGACUACCCUAUCAAGUAATT |
|  | Anti-sense | UUACUUGAUAGGGUAGUCCTT |
| si*CREBBP* | Sense | UCAACUCCUGUGUCGUCUUTT |
|  | Anti-sense | AAGACGACACAGGAGUUGATT |
| si*KAT2B* | Sense | CCACCAUGAGUGGUGUCUATT |
|  | Anti-sense | UAGACACCACUCAUGGUGGTT |
| si*STAT1* | Sense | CCCUGAAGUAUCUGUAUCCAA |
|  | Anti-sense | UUGGAUACAGAUACUUCAGGG |
| si*STAT3* | Sense | GCAAAGAAUCACAUGCCACUU |
|  | Anti-sense | AAGUGGCAUGUGAUUCUUUGC |

Table S3 The primers for q-RT-PCR

| Gene name | | Primer Sequence (5’→3’) |
| --- | --- | --- |
| *ACTIN* | Forward | ATTCCTATGTGGGCGACGAG |
|  | Reverse | CCAGATTTTCTCCATGTCGTCC |
| *PD-L1* | Forward | CCCTAATTTGAGGGTCAGTTCCT |
|  | Reverse | CTCAGTCATGCAGAAAACAAATTGA |
| *KLF12* | Forward | AAGGGAGACCCTCCTAATGAGA |
|  | Reverse | GTGTCTGAGTCAGTTAGGGAGTT |
| *TCF7* | Forward | AGCACCAAGAATCCACCACA |
|  | Reverse | GTCACTTTCAGGGGGTAGGC |
| *ZNF703* | Forward | TCTAACCCAAGGACACCCGA |
|  | Reverse | TTCTTGGCGTCCAGCTCAAT |
| *ZNF623* | Forward | AGGGCACAGTCCGCCG |
|  | Reverse | TTAGAATACGGCGCTCCTCAG |
| *Gapdh* | Forward | ACCACAGTCCATGCCATCAC |
|  | Reverse | TCCACCACCCTGTTGCTGTA |
| *Klf8* | Forward | CAAGCCATTATGGTGCCTAC |
|  | Reverse | ATAGAGCCCGGAGTGAGAAC |
| *PD-L1* Promoter (STAT1/3 bindng site) | Forward | TGGACTGACATGTTTCACTTTCT |
|  | Reverse | CAAGGCAGCAAATCCAGTTT |
| *PD-L1* Promoter  (KLF12 bindng site) #1 | Forward | CGAGGAACTTTGAGGAAGTCACAG |
|  | Reverse | AAAGTCAGCAGCAGACCCATATGG |
| *PD-L1* Promoter  (KLF12 binding site) #2 | Forward | CCTAGAGGTCACAGTCACCAAAGT |
|  | Reverse | GTTTGGGCCCAGCTCAGATGTT |

Table S4 Clinicopathologic characteristics of 33 clinical lung cancer patient tissues

| **Patient No.** | **Gender** | **Age** | **Histotype** | **TNM** | **Genetic testing** |
| --- | --- | --- | --- | --- | --- |
| 1 | male | 74 | LUSC | T1N3M0 | / |
| 2 | female | 80 | others | T4N0M1 | AKT amplification; PTEN, and RB 1 deletion |
| 3 | male | 73 | LUSC | T4N0M0 | / |
| 4 | female | 70 | LUAD | T2bN2M0 | ALK(D5F3)(-), ALK-NC(-), ROS1(+~++,20%), c-Met(+~++,10%), CK5/6(-), Napsin A(+), P40(-), P63(-), TTF1(+), CK7(+) |
| 5 | female | 65 | LUAD | T2N0M0 | ALK(D5F3)(-), ALK-NC(-), CK5/6(-), TTF1(+), Napsin A(+), P63(+), CK7(+) |
| 6 | male | 60 | others | / | / |
| 7 | male | 70 | others | T4N0M0 | negtive |
| 8 | male | 69 | LUSC | T2N0M0 | ALK(D5F3)(-), ALK-NC(-), CK5/6(+), CK7(+), Napsin A(-), P63(+), TTF1(-) |
| 9 | male | 66 | LUSC | T4N2M0 | negtive |
| 10 | female | 68 | LUAD | T2N0M0 | ALK(D5F3)(-), ALK-NC(-), CK5/6(-), CK7(+), Napsin A(+), P63(+), TTF1(+) |
| 11 | male | 71 | LUSC | T2N3M0 | / |
| 12 | male | 72 | LUAD | T3N0M0 | The EGFR Ex19 Del mutation |
| 13 | male | 75 | LUSC | T2N0M0 | ALK(D5F3)(-), ALK-NC(-), CK5/6(+), CK7(-), Napsin A(-), P63(+), TTF1(-) |
| 14 | male | 67 | LUSC | T2N1M0 | ALK(D5F3)(+), ALK-NC(-), CK5/6(+), CK7(-), Napsin A(-), P63(+), TTF1(-) |
| 15 | male | 76 | LUAD | T2N1M0 | ALK(D5F3)(-), ALK-NC(-), CK5/6(+), CK7(+), Napsin A(+), P63(+), TTF1(+) |
| 16 | female | 76 | LUAD | T4N0M0 | ALK(D5F3)(-), ALK-NC(-), CK5/6(-), Napsin A(+), P40(-), P63(+), TTF1(+), CK7(+) |
| 17 | male | 77 | LUAD | T2aN0M0 | The EGFR Ex21 L858R mutation |
| 18 | male | 64 | others | / | / |
| 19 | female | 63 | LUAD | T4N0M0 | ALK(D5F3)(+), ALK-NC(-), CK5/6(-), Napsin A(+), P40(-), P63(+), TTF1(+), CK7(+) |
| 20 | female | 85 | LUAD | T2N0M0 | ALK(D5F3)(-), ALK-NC(-), ROS1(+), c-Met(+), CK5/6(-), Napsin A(+), P40(-), P63(-), TTF1(+), CK7(+) |
| 21 | female | 70 | LUAD | T4N0M0 | ALK-NC(-), ALK(D5F3)(-), TTF1(-), Napsin A(-), hMAM\Mammaglobin(-), GCDFP-15(-), GATA-3(-) |
| 22 | female | 61 | LUAD | T2N0M0 | ALK(D5F3)(-), ALK-NC(-), ROS1(-), c-Met(+), CK7(+), Napsin A(+), P40(-), P63(-), TTF1(+), CK5/6(-) |
| 23 | male | 71 | LUSC | T1N0M0 | ALK(D5F3)(-), ALK-NC(-), ROS1(-), c-Met(-), CK5/6(+), Napsin A(-), P40(+), P63(+), TTF1(-), CK7(-) |
| 24 | male | 70 | LUAD | T3N1M0 | ALK(D5F3)(-), ROS1(-), c-Met(-), CK7(+), Napsin A(+), P40(-), P63(-), TTF1(+), CK5/6(-) |
| 25 | male | 74 | LUAD | T2N0M0 | ALK(D5F3)(-), ROS1(-), c-Met(+), CK5/6(-), Napsin A(+), P40(-), P63(-), TTF1(+), CK7(+), the EGFR Ex20 Ins mutation |
| 26 | female | 66 | others | / | / |
| 27 | male | 75 | LUSC | T2N1M0 | ALK(D5F3)(-), ROS1(-), c-Met(-), CK5/6(+), CK7(-), P40(+), P63(+), TTF1(-), Napsin A(+) |
| 28 | male | 66 | LUSC | T2N0M0 | ALK(D5F3)(-), ROS1(-), c-Met(-), CK5/6(+), CK7(-), P40(+), P63(+), TTF1(-), Napsin A(-) |
| 29 | male | 66 | LUSC | T4N0M0 | CK5/6(+), Napsin A(-), P40(+), P63(+), c-Met(+), CK7(+), ALK(D5F3)(-), ROS1(-), TTF1(-) |
| 30 | male | 43 | LUSC | / | / |
| 31 | male | 62 | LUSC | / | / |
| 32 | female | 76 | LUAD | / | / |
| 33 | male | 64 | LUSC | / | / |

LUSC: Lung squamous cell carcinoma; LUAD: Lung adenocarcinoma; TNM stage based on The 8th Edition Lung CancerStage Classification; All lung cancer patients have not been performed with immune checkpoints inhibitors.

Table S5 Clinicopathologic characteristics of lung cancer tissues microarray cohorts

| **Patient No.** | **Gender** | **Age** | **Histotype** | **TNM** | **PFS**  **(months)** | **OS**  **(months)** |
| --- | --- | --- | --- | --- | --- | --- |
| 1 | male | 54 | others | T2N0M0 | 0 | 75 |
| 2 | male | 68 | LUSC | T2N2M0 | 15 | 17 |
| 3 | male | 70 | LUSC | T3N0M0 | 10 | 29 |
| 4 | female | 57 | LUAD | T2N0M0 | 10 | 24 |
| 5 | male | 50 | LUSC | T2N0M0 | 71 | 71 |
| 6 | male | 51 | LUAD | T4N0M0 | 107 | 107 |
| 7 | male | 62 | others | T2N2M0 | 69 | 70 |
| 8 | female | 49 | LUAD | T4N2M1 | 25 | 29 |
| 9 | male | 61 | LUSC | T2N2M0 | 0 | 34 |
| 10 | male | 65 | others | T2N0M0 | 15 | 20 |
| 11 | male | 51 | LUSC | T2N1M0 | 92 | 92 |
| 12 | male | 55 | LUSC | T3N1M0 | 86 | 105 |
| 13 | female | 61 | LUAD | T2N1M0 | 31 | 34 |
| 14 | male | 48 | LUSC | T2N0M0 | 68 | 69 |
| 15 | female | 48 | LUAD | T2N0M0 | 91 | 91 |
| 16 | female | 64 | LUAD | T1N0M0 | 91 | 91 |
| 17 | male | 72 | LUAD | T2N1M0 | 78 | 78 |
| 18 | female | 60 | LUAD | T2N1M0 | 25 | 54 |
| 19 | male | 66 | LUSC | T2N0M0 | 90 | 90 |
| 20 | female | 54 | LUAD | T2N0M0 | 13 | 90 |
| 21 | female | 57 | LUAD | T2N0M0 | 77 | 77 |
| 22 | male | 58 | LUAD | T2N0M0 | 90 | 90 |
| 23 | male | 61 | LUSC | T2N0M0 | 40 | 90 |
| 24 | male | 63 | LUSC | T2N0M0 | 90 | 90 |
| 25 | male | 62 | LUSC | T2N0M0 | 53 | 53 |
| 26 | male | 70 | LUSC | T2N1M0 | 89 | 89 |
| 27 | male | 64 | SCLC | T2N0M0 | 89 | 89 |
| 28 | male | 73 | LUSC | T2N0M0 | 89 | 89 |
| 29 | female | 71 | LUAD | T2N0M0 | 89 | 89 |
| 30 | male | 60 | LUAD | T2N0M0 | 89 | 52 |
| 31 | male | 75 | LUSC | T3N0M0 | 2 | 4 |
| 32 | male | 52 | LUSC | T3N2M0 | 52 | 12 |
| 33 | male | 73 | LUAD | T2N0M0 | 76 | 59 |
| 34 | male | 69 | LUSC | T2N0M0 | 76 | 76 |
| 35 | female | 46 | LUSC | T3N2M0 | 8 | 52 |
| 36 | female | 54 | LUAD | T2N0M0 | 89 | 89 |
| 37 | male | 53 | LUSC | T4N1M0 | 52 | 5 |
| 38 | male | 56 | LUSC | T4N2M0 | 52 | 4 |
| 39 | male | 75 | LUSC | T1N0M0 | 19 | 71 |
| 40 | male | 43 | LUSC | T2N1M0 | 88 | 88 |
| 41 | female | 45 | LUAD | T1N0M0 | 88 | 88 |
| 42 | male | 67 | LUAD | T2N0M0 | 51 | 51 |
| 43 | female | 65 | LUAD | T2N0M0 | 88 | 88 |
| 44 | male | 54 | LUSC | T4N1M0 | 74 | 3 |
| 45 | male | 58 | LUSC | T2N1M0 | 4 | 48 |
| 46 | female | 60 | LUAD | T1N0M0 | 87 | 87 |
| 47 | male | 64 | LUAD | T2N2M0 | 50 | 50 |
| 48 | female | 67 | LUAD | T2N0M0 | 50 | 51 |
| 49 | male | 55 | LUAD | T1N0M0 | 74 | 74 |
| 50 | female | 63 | LUAD | T1N2M0 | 50 | 51 |
| 51 | male | 49 | SCLC | T2N2M0 | 87 | 87 |
| 52 | male | 56 | LUSC | T4N1M0 | 87 | 87 |
| 53 | female | 43 | LUAD | T1N0M0 | 50 | 50 |
| 54 | male | 58 | LUAD | T4N2M0 | 37 | 87 |
| 55 | male | 70 | LUAD | T1N0M0 | 15 | 74 |
| 56 | male | 74 | LUAD | T2N0M0 | 5 | 10 |
| 57 | female | 51 | LUSC | T2N1M0 | 86 | 86 |
| 58 | male | 62 | LUSC | T2N1M0 | 49 | 41 |
| 59 | male | 65 | LUAD | T2N0M0 | 37 | 76 |
| 60 | male | 56 | LUSC | T3N0M0 | 49 | 49 |
| 61 | male | 60 | LUAD | T4N1M0 | 9 | 42 |
| 62 | male | 74 | LUAD | T2N0M0 | 19 | 41 |
| 63 | male | 53 | LUSC | T3N1M0 | 0 | 48 |
| 64 | male | 55 | LUSC | T4N2M0 | 85 | 85 |
| 65 | male | 67 | LUSC | T3N1M0 | 4 | 14 |
| 66 | male | 58 | LUSC | T2N1M0 | 48 | 18 |
| 67 | male | 41 | LUSC | T4N1M0 | 48 | 48 |
| 68 | male | 63 | LUSC | T2N0M0 | 72 | 72 |
| 69 | male | 43 | LUSC | T2N2M0 | 48 | 48 |
| 70 | male | 70 | LUSC | T2N0M0 | 37 | 42 |
| 71 | male | 67 | LUSC | T2N1M0 | 85 | 85 |
| 72 | male | 50 | LUAD | T1N0M0 | 84 | 84 |
| 73 | male | 66 | LUSC | T3N1M0 | 2 | 71 |
| 74 | female | 60 | LUAD | T2N0M0 | 9 | 72 |
| 75 | male | 66 | LUAD | T2N0M0 | 84 | 84 |
| 76 | male | 40 | LUAD | T2N2M0 | 2 | 33 |
| 77 | male | 56 | LUSC | T2N0M0 | 71 | 71 |
| 78 | male | 69 | LUSC | T3N0M0 | 52 | 53 |
| 79 | male | 67 | LUSC | T2N2M0 | 76 | 76 |
| 80 | female | 65 | LUAD | T1N2M0 | 76 | 76 |
| 81 | male | 56 | LUAD | T2N1M0 | 76 | 76 |
| 82 | male | 68 | SCLC | T1N0M0 | 39 | 40 |
| 83 | female | 73 | LUSC | T1N0M0 | 76 | 76 |
| 84 | male | 60 | LUSC | T2N1M0 | 39 | 37 |
| 85 | male | 68 | LUSC | T2N0M0 | 75 | 75 |
| 86 | female | 45 | LUAD | T2N1M0 | 45 | 51 |
| 87 | female | 72 | LUAD | T2N0M0 | 38 | 39 |
| 88 | male | 57 | LUSC | T2N0M0 | 75 | 75 |
| 89 | male | 55 | LUSC | T2N0M0 | 62 | 62 |
| 90 | male | 63 | LUAD | T2N2M0 | 75 | 75 |
| 91 | female | 63 | LUAD | T1N0M0 | 75 | 75 |
| 92 | male | 65 | LUSC | T3N1M0 | 75 | 75 |
| 93 | male | 64 | LUAD | T2N2M0 | 38 | 39 |
| 94 | female | 62 | LUAD | T2N0M0 | 38 | 31 |
| 95 | male | 64 | LUSC | T2N1M0 | 75 | 75 |
| 96 | male | 71 | LUAD | T2N0M0 | 74 | 74 |
| 97 | male | 74 | LUSC | T2N0M0 | 61 | 61 |
| 98 | female | 59 | LUAD | T2N0M0 | 61 | 61 |
| 99 | female | 63 | others | T1N2M0 | 37 | 61 |
| 100 | male | 53 | LUAD | T3N0M0 | 37 | 37 |
| 101 | male | 56 | LUSC | T2N0M0 | 74 | 74 |
| 102 | male | 53 | LUSC | T2N1M0 | 74 | 74 |
| 103 | male | 66 | LUAD | T2N2M0 | 61 | 61 |
| 104 | female | 49 | others | T1N0M0 | 74 | 74 |
| 105 | male | 61 | LUSC | T1N0M0 | 37 | 37 |
| 106 | male | 53 | LUSC | T2N0M0 | 73 | 73 |
| 107 | male | 53 | LUAD | T4N0M0 | 19 | 29 |
| 108 | female | 61 | LUAD | T2N0M0 | 36 | 37 |
| 109 | female | 62 | LUAD | T2N0M0 | 60 | 60 |
| 110 | male | 66 | LUSC | T2N0M0 | 60 | 60 |

PFS, progression-free survival; OS, overall survival; LUSC: Lung squamous cell carcinoma; LUAD: Lung adenocarcinoma; TNM stage based on The 8th Edition Lung CancerStage Classification; All lung cancer patients have not been performed with immune checkpoints inhibitors
